# Global, regional and national burdens of cardiovascular disease attributable to secondhand smoke from 1990–2019: an age-period-cohort analysis

**DOI:** 10.1136/openhrt-2024-003079

**Published:** 2025-02-11

**Authors:** Hong Jiang, Zeye Liu, Peijian Wei, Fengwen Zhang, Shouzheng Wang, Wen-Bin Ou-yang, Xiaofei Li, Xiang-Bin Pan

**Affiliations:** 1 Department of Structural Heart Disease, Fuwai Hospital, Chinese Academy of Medical Sciences & Peking Union Medical College, Beijing, People's Republic of China; 2 Department of Cardiac Surgery, Peking University People's Hospital & Peking University, Beijing, People's Republic of China; 3 Department of Cardiology, Chinese Academy of Medical Sciences & Peking Union Medical College Fuwai Hospital, Xicheng District, Beijing, China

**Keywords:** Smoking, Stroke, Death, Sudden, Cardiac

## Abstract

**Background:**

Over the past three decades, significant disparities in the global burden of cardiovascular disease (CVD) have been observed, particularly CVD attributed to secondhand smoke. However, a comprehensive understanding of global trends and their interaction with secondhand smoke remains inadequate.

**Methods:**

Using Global Burden of Disease data (1990–2019), an age-period-cohort analysis examined temporal trends in CVD mortality among secondhand smoke-exposed populations, considering age, period and cohort interactions.

**Results:**

Over the 30-year period, the global number of CVD deaths attributed to secondhand smoke increased substantially, from 432.6 thousand in 1990 (95% UI: 357.4–508.3) to 598.5 thousand in 2019 (95% UI: 489.7–713.5), representing a 38.4% increase (95% UI: 26.8%–49.5%). In 2019, CVD accounted for 45.9% of all deaths attributable to secondhand smoke among both sexes globally. Among these CVD deaths, ischaemic heart disease predominated, accounting for 66.4% of cases, compared with stroke. The distribution by sex revealed a slightly lower percentage of males (46.5%) than females (53.5%). Age-period-cohort models show overall global decline in CVD mortality due to secondhand smoke over 30 years, with regional, sex and subtype variations. Notably, a higher Sociodemographic Index (SDI) correlated with a greater reduction in mortality, exhibiting a significant 39.1% decrease in high SDI areas (95% UI: 35.6%–42.3%), in stark contrast to the minimal change observed in low SDI areas (0.1%, 95% UI: −52.4%–62.2%).

**Conclusions:**

This study highlights the importance of considering secondhand smoke as a modifiable CVD risk. Further research is needed to understand disparities in CVD burden across development levels, sexes and subtypes.

**WHAT IS ALREADY KNOWN ON THIS TOPIC**

- Cardiovascular disease (CVD) stands as a primary contributor to global mortality, exhibiting considerable disparities in its burden across the globe. Notably, secondhand smoke has been firmly established as a pivotal risk factor for CVD and other significant non-communicable diseases. Furthermore, the number of CVD deaths attributed to secondhand smoke has consistently risen, primarily due to population expansion and the ageing demographic. However, there persists a gap in our comprehension of the comprehensive and dynamic nature of the global CVD burden attributable to secondhand smoke.

**WHAT THIS STUDY ADDS**

- This research offers an in-depth longitudinal analysis of global CVD-related mortality attributable to secondhand smoke, employing the age-period-cohort model and drawing on data from the Global Burden of Disease 2019. The study delves into identifying trends in the burden of CVD and variations in the prevalence of secondhand smoke over the span of three decades. Additionally, it scrutinises the interplay between socioeconomic development, gauged by the Sociodemographic Index, and the influence of secondhand smoke on the global CVD burden.

**HOW THIS STUDY MIGHT AFFECT RESEARCH, PRACTICE OR POLICY**

- The findings highlight the global disparity between age-standardised and all-age mortality from secondhand smoke, underscoring the importance of considering the age composition of CVD-related mortality in different regions. In addition, the study highlights the need for targeted public health interventions and policies to control secondhand smoke exposure and reduce cardiovascular disease-related mortality in the region, especially given the region’s ageing population. Additionally, insights into variations in CVD burden based on socioeconomic development are provided, indicating that countries with lower development indices experience higher age-standardised mortality rates from CVD attributable to secondhand smoke. These results have significant implications for resource allocation, programme planning and evidence-based strategies to improve cardiovascular health globally. Nonetheless, the study acknowledges the limitations of data quality and emphasises the need for further research to comprehend inter-country variations and sex disparities in CVD subtypes.

## Introduction

 Studies have shown that cardiovascular disease (CVD) has multiple risk factors. Effective control can notably reduce incidence and mortality.^1^ Secondhand smoke is a crucial yet overlooked CVD risk factor. Global burden of disease (GBD) data indicate that in 2019, it caused over 1.3 million deaths worldwide, with 590 000 (45%) due to CVD, including ischaemic heart disease (30%) and stroke (15%).^2^

As early as 1974, two studies published in The Lancet indicated that infants of parents who smoked had a higher risk of developing pneumonia and bronchiolitis.^3^ Since then, people have become increasingly aware of the potential health risks of secondhand smoke. A systematic review reveals that ischaemic heart disease and stroke are strong evidence of causation with secondhand smoke.^4^

It is essential to realise that tobacco control is the key to achieve major wins for public health and save economies billions in healthcare and productivity costs.^5^ Thus, this study analysed the distribution of and changes in the global disease burden of CVD attributable to secondhand smoke between 1990 and 2019 using the public databases of the GBD, Injuries, and Risk Factors Study 2019.

## Methods

### Data sources

The GBD is an ongoing international collaboration research project, detailed protocols for GBD 2019 are available through the website of Institute for Health Metrics and Evaluation.^6^ Our study obtained the data from the GBD 2019 public datasets, available from https://vizhub.healthdata.org/gbd-results/. Despite the release of GBD 2021, we have elected to use the data from GBD 2019, considering the significant impact of the COVID-19 pandemic on the disease burden, particularly on cardiovascular diseases, which has disrupted the normal trend estimation of secondhand smoke. Our objective is to align our research with disease burden estimates under normalised conditions, contributing to health policy formulation in such circumstances. The study adhered to the principles of the Declaration of Helsinki regarding ethical considerations. Due to the use of de-identified aggregated data, the University of Washington Institutional Review Board approved the waiver of informed consent.

### Definitions

The GBD 2019 followed an overarching conceptual framework established for comparative risk assessment (CRA).^7^ The GBD CRA introduces a hierarchy containing four levels to classify risks.^8^ The hierarchical classification of causes of diseases and injuries in the GBD 2019 consists of four levels.^9^ In our study, secondhand smoke (a level 3 risk) was defined according to the GBD 2019 definition. We analysed 30-year trends (1990–2019) in age-standardised mortality rates and age-group shifts in mortality (six categories), calculating mortality ratios per group and age-adjusted rates based on GBD 2019 data.^10^

For our study, secondhand smoke (level 3 risk) was defined using the GBD 2019 definition. This risk factor pertains to current exposure to secondhand tobacco smoke within domestic, occupational or communal settings. In terms of the consideration of exposed populations, GBD 2019 considers that only non-daily smokers are deemed to be exposed to secondhand smoke.^11^ According to the definition of the GBD 2019, cardiovascular diseases encompass both atherosclerotic and non-atherosclerotic cardiovascular diseases and include death and disability resulting from 11 cardiovascular causes.^12^

Based on total fertility rate among females younger than 25 years, educational attainment for those aged 15 years or older, and lag-distributed income per capita,^7 8 13^ a composite indicator of a geographical location’s development status, known as Sociodemographic Index (SDI), were calculated.^14^

### Statistical analysis

In order to access the potential mortality trends among different birth cohorts, time periods and age brackets, a commonly employed statistical technique named the age-period-cohort (APC) model was employed.^15^ This study used APC regression to assess age, period and cohort effects on CVD mortality trends, exploring long-term trends and identifying factors (biological, social, environmental) contributing to the disease.^15 16^

In this study, the input data for the APC model include population data and mortality rates of individuals affected by secondhand smoke from various countries and regions between 1990 and 2019. Before inputting data into the APC model, the data is processed to generate GBD (Global Burden of Disease) data in equally spaced intervals, conforming to the model’s conditions of consistent age, period and cohort intervals. To display long-term trends, the observation period is extended as much as possible. Population and mortality data span from 1990 to 2019, segmented into consecutive 5-year intervals. Similarly, age groups and birth cohorts are categorised into consecutive 5-year intervals (online supplemental figure S1). The APC model used in this study employs a logarithmic function based on the Poisson distribution, capable of estimating cumulative effects caused by changes in age, period and birth cohort. Through the APC model, net drift can be estimated, which refers to the adjusted change in the logarithm of the overall mortality rate after accounting for non-linear period and birth cohort effects. This can be understood as the estimated annual percentage change in age-standardised rates over time.^17 18^

Finally, we delved into the trends of cardiovascular disease burden attributable to secondhand smoke from 1990 to 2019 using gender-stratified Joinpoint regression models. To accurately estimate changes in incidence rates, we employed the least squares method, thereby avoiding subjective influences that traditional trend analyses based on linear models may introduce.^19^

Subsequently, we calculated annual percentage changes and their 95% CI at Joinpoint change points. We then constructed a baseline model to determine average annual percentage changes (AAPC) and their 95% CI for the entire study period (1990–2019) and the recent decade (2010–2019). The AAPC, a weighted average of APCs based on stage length, provides a comprehensive measure of trend variations.^20^

For statistical testing, we used the Wald χ² test to assess the significance of relevant parameters and functions, with a two-sided significance level of p<0.05. For data analysis, we employed R software version 3.6.3 and Joinpoint Regression Programme version 4.9.1.0 for Joinpoint regression modelling and trend analysis.

## Results

### Global trends in cardiovascular disease-related mortality burden attributable to secondhand smoke

In 2019, global CVD deaths due to secondhand smoke totaled 598.5K, with an age-standardised rate of 7.4/100K (table 1). Despite a 35.1% decline in age-standardised CVD mortality rates, absolute deaths rose 38.4% (95% UI: 26.8%–49.5%) (table 1). Secondhand smoke’s proportion of overall CVD mortality remained stable, marginally decreasing from 3.2% to 3.1% (table 1).

**Table 1:** Cardiovascular mortality attributable to secondhand smoke risk by sex from 1990 to 2019

Locations	Variables	Both sexes			Female			Male		
		1990	2019	Change, %	1990	2019	Change, %	1990	2019	Change, %
Global	Deaths, n	432 558.3 (357 386.1, 508 293.6)	598 474.7 (489 749.3, 713 482.2)	38.4 (26.8, 49.5)	241 044.4 (199 540.9, 286 844.76)	320 041.7 (261 416.7, 384 510.5)	32.8 (16.7, 48.2)	191 513.9 (156 605.3, 225 486.4)	278 433.1 (224 397.9, 337 392.9)	45.4 (30.6, 61.9)
	ASMR, per 100 000	11.4 (9.5, 13.4)	7.4 (6.1, 8.9)	−35.1 (–40.3, –30.3)	11.6 (9.6, 13.7)	7.3 (6.0, 8.8)	−37.0 (–44.4, –30.0)	11.1 (9.1, 13.1)	7.5 (6.1, 9.1)	−32.6 (–38.9, –25.4)
	Proportion, %	3.2 (2.7, 3.8)	3.1 (2.6, 3.8)	−4.0 (–7.8, –0.4)	3.7 (3.1, 4.3)	3.6 (3.0, 4.2)	−3.0 (–8.1, 1.9)	2.8 (2.3, 3.2)	2.7 (2.2, 3.2)	−3.5 (–8.8, 2.0)
African Region	Deaths, n	13 685.0 (10 784.9, 16 775.6)	22 343.82 (17 332.44, 27 713.69)	63.3 (41.0, 87.2)	7024.77 (5550.67, 8645.80)	11 716.7 (9076.0, 14 590.7)	66.8 (41.3, 95.2)	6660.2 (5150.3, 8227.2)	10 627.1 (8121.7, 13 286.9)	59.6 (36.4, 67.4)
	ASMR, per 100 000	6.5 (5.2, 8.0)	5.0 (3.9, 6.2)	−23.6 (−33.6, –13.1)	6.5 (5.1, 8.1)	5.0 (3.8, 6.2)	−24.3 (−35.8, –12.2)	6.5 (5.1, 8.0)	5.0 (3.9, 6.3)	−22.7 (−33.6, –10.5)
	Proportion, %	2.0 (1.6, 2.4)	1.8 (1.4, 2.2)	−9.6 (−14.8, –4.5)	2.1 (1.7, 2.5)	1.9 (1.5, 2.2)	−11.5 (−18.0, –4.5)	1.9 (1.5, 2.3)	1.8 (1.4, 2.1)	−7.8 (−13.5, –1.5)
Eastern Mediterranean Region	Deaths, n	34 960.7 (28 361.0, 41 142.7)	61 899.2 (49 456.2 74 429.6)	77.1 (55.2, 102.2)	18 198.62 (14 867.85, 21 531.42)	31 268.6 (24 823.2, 37 783.8)	71.8 (45.8, 101.1)	16 762.1 (13 476.5, 19 864.1)	30 630.5 (24 149.1, 37 442.0)	82.7 (60.9, 107.5)
	ASMR, per 100 000	20.0 (16.3, 23.6)	15.2 (12.3, 18.3)	−23.7 (−32.5, –13.8)	21.8 (17.7, 25.8)	16.1 (12.9, 19.4)	−26.3 (−36.6, –14.2)	18.3 (14.8, 21.7)	14.5 (11.6, 17.4)	−20.7 (−29.6, –10.7)
	Proportion, %	4.3 (3.6, 5.0)	3.9 (3.2, 4.5)	−10.5 (−13.9, –7.0)	4.8 (4.1, 5.6)	4.3 (3.6, 5.0)	−12.0 (−16.5, –7.3)	3.9 (3.2, 4.5)	3.5 (2.9, 4.2)	−8.7 (−12.9, –4.7)
European Region	Deaths, n	123 577.0 (101 792.2, 144 952.3)	99 019.8 (82 081.4, 116 606.0)	−19.9 (−14.1, –25.7)	69 412.1 (56 547.2, 81 908.4)	54 484.0 (44 592.5, 65 115.3)	−21.5 (−28.2, –14.2)	98 853.9 (74 033.0, 127 205.4)	44 535.9 (36 492.2, 52 838.0)	−17.8 (−24.6, –10.2)
	ASMR, per 100 000	11.7 (9.7, 13.6)	6.1 (5.1, 7.2)	−47.4, (−51.1, –43.9)	10.5 (8.7, 12.3)	5.6 (4.6, 6.6)	−46.9 (−51.3, –42.3)	12.8 (10.7, 15.0)	6.6 (5.5, 7.8)	−48.3 (−52.4, –44.1)
	Proportion, %	3.0 (2.5, 3.4)	2.5 (2.1, 3.0)	−14.5 (−17.6, –11.1)	3.2 (2.7, 3.7)	2.7 (2.3, 3.2)	−12.9 (−17.2, –8.6)	2.7 (2.3, 3.1)	2.3 (1.9, 2.7)	−13.6 (−18.2, –8.7)
Region of the Americas	Deaths, n	76 204.3 (60 951.3, 92 071.8)	39 890.7 (32 471.4, 47 426.1)	−18.4 (−23.4, –12.9)	18 963.1 (15 558.4, 22 222.4)	15 806.65 (12 747.05, 18 885.59)	−16.6 (−22.8, –10.6)	29 902.7 (24 646.1, 35 163.5)	24 084.1 (19 573.5, 29 027.7)	−19.5 (−26.0, –12.2)
	ASMR, per 100 000	8.1 (6.7, 9.4)	3.1 (2.6, 3.7)	−61.2 (−63.5, –58.6)	5.6 (4.6, 6.5)	2.2 (1.8, 2.7)	−59.9 (−62.7, –57.0)	11.1 (9.2, 13.0)	4.2 (3.4, 5.0)	−62.3 (−65.3, –59.1)
	Proportion, %	2.9 (2.4, 3.4)	1.9 (1.6, 2.3)	−33.5 (−36.5, –30.4)	2.4 (2.0, 2.8)	1.7 (1.4, 2.0)	−31.6 (−35.5, –28.0)	3.3 (2.7, 3.9)	2.2 (1.8, 2.6)	−34.6 (−38.7, –30.4)
South-East Asia Region	Deaths, n	76 204.3 (60 951.3, 92 071.8)	152 973.2 (122 153.9, 185 582.7)	100.7 (74.3, 127.3)	43 151.4 (33 910.5, 52 828.7)	83 928.4 (65 174.5, 104 318.7)	94.5 (60.4, 32.0)	33 053.0 (25 984.8, 40 338.7)	69 044.8 (53 127.1, 87 034.7)	108.9 (72.9, 153.4)
	ASMR, per 100 000	11.9 (9.6, 14.4)	9.3 (7.4, 11.3)	−21.9 (−32.0, –11.5)	13.5 (10.7, 16.7)	9.8 (7.6, 12.2)	−27.7 (−40.4, –13.7)	10.2 (8.1, 12.4)	8.7 (6.7, 10.9)	−14.4 (−28.7, 2.8)
	Proportion, %	3.6 (2.9, 4.2)	3.5 (2.8, 4.1)	−2.1 (−7.6, 3.8)	4.5 (3.7, 5.3)	4.2 (3.4, 4.9)	−7.2 (−13.0, –0.3)	2.8 (2.2, 3.3)	2.9 (2.2, 3.3)	4.2 (−5.4, 14.4)
Western Pacific Region	Deaths, n	134 288.2 (106 348.7, 163 750.1)	221 036.3 (174 180.4, 269 542.9)	64.6 (36.9, 97.3)	83 784.9 (65 025.9, 103 490.1)	122 182.5 (94 923.6, 155 716.5)	45.8 (13.0, 84.7)	50 503.3 (38 734.7, 63 453.2)	98 853.9 (74 033.0, 127 205.4)	95.7 (51.5, 153.9)
	ASMR, per 100 000	12.7 (10.1, 15.5)	8.4 (6.7, 10.3)	−33.7 (−44.6, –21.4)	14.6 (11.5, 18.0)	8.5 (6.6, 10.8)	−41.8 (−54.4, –27.2)	10.4 (8.1, 2.8)	8.3 (6.3, 10.6)	−20.4 (−37.5, 1.9)
	Proportion, %	3.8 (3.1, 4.5)	3.7 (3.0, 4.4)	−1.8 (−10.1, 7.0)	4.8 (4.0, 5.7)	4.7 (3.9, 5.6)	−2.5 (−11.1, 6.3)	2.7 (2.2, 3.3)	2.9 (2.3, 3.5)	7.0 (−7.3, 23.3)

Data in parentheses are 95% uncertainty intervals.

ASMR, age-standardised mortality rate

**Figure 1:**
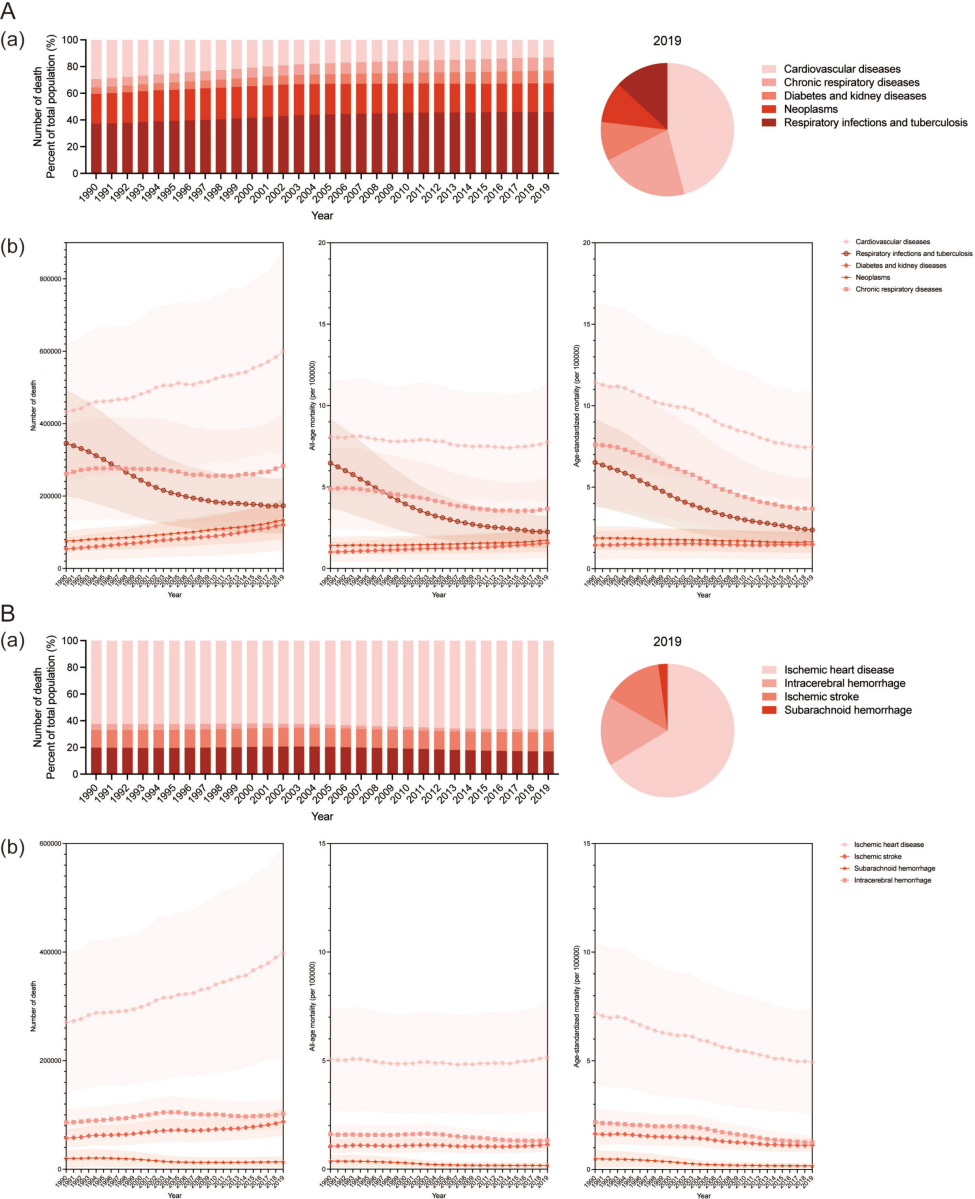
The composition of attributable deaths globally from 1990 to 2019 for both sexes combined encompasses the composition of deaths attributable to cardiovascular diseases caused by secondhand smoke. A(a) Composition of attributable deaths from 1990 to 2019. (**b**) Temporal trends in the composition of deaths attributable from 1990 to 2019. The solid lines and shaded areas indicate the number or rate of deaths and their respective 95% uncertainty intervals. B(a) Composition of attributable CVD deaths from 1990 to 2019. (**b**) Temporal trends in the composition of CVD deaths attributable from 1990 to 2019. The solid lines and shaded areas indicate the number or rate of deaths and their respective 95% uncertainty intervals. CVD, cardiovascular disease.

**Figure 2:**
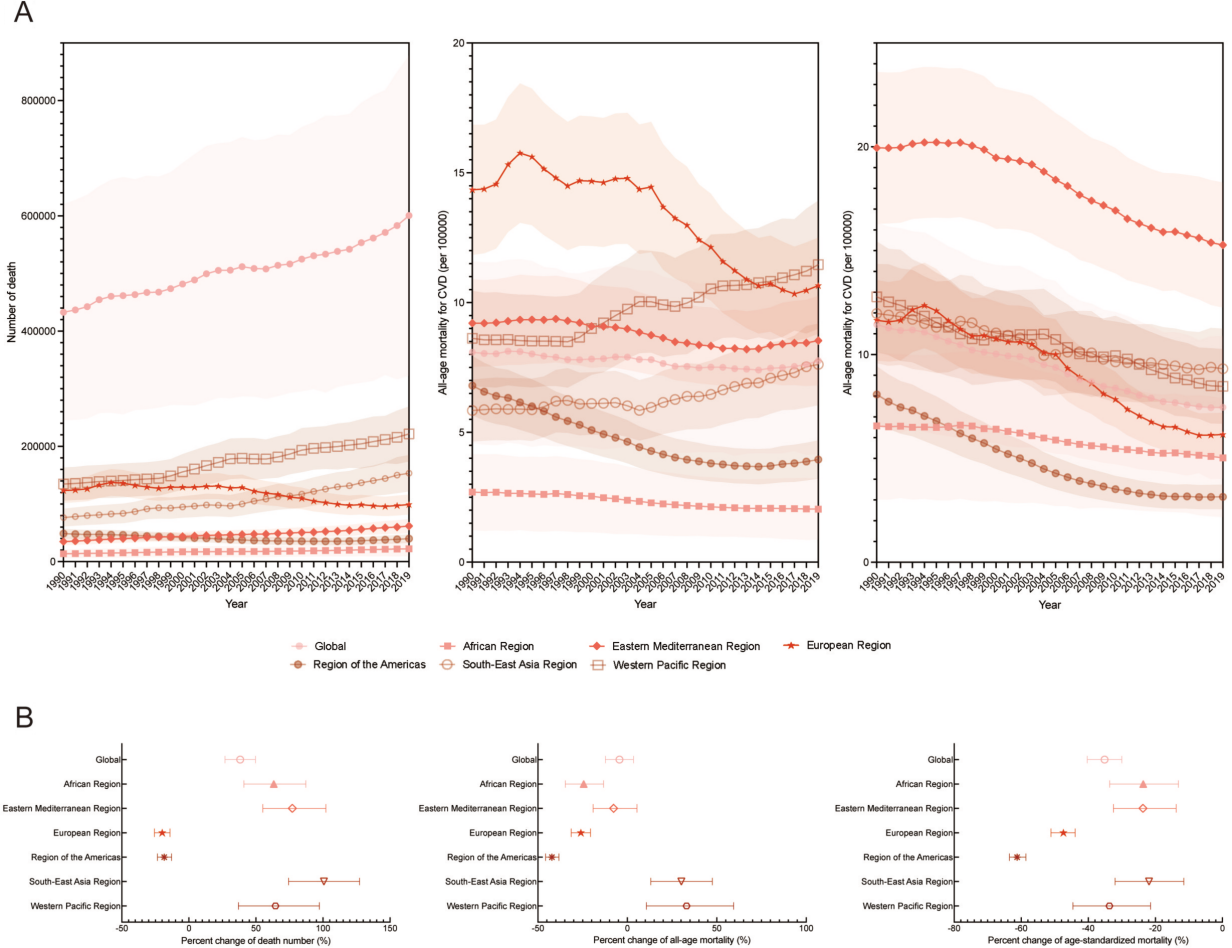
Temporal trends in the number of deaths, all-age mortality and age-standardised mortality attributed to CVD caused by secondhand smoke for both sexes combined across the six WHO regions from 1990 to 2019. (**A**) Temporal trends in the number of deaths, all-age mortality and age-standardised mortality due to CVD from 1990 to 2019. The solid lines and shaded areas indicate the number or rate of deaths and their respective 95% uncertainty intervals. (**B**) Percentage change in the number of deaths, all-age mortality and age-standardised mortality attributed to CVD caused by secondhand smoke across the six WHO regions from 1990 to 2019. CVD, cardiovascular disease.

In 2019, CVD accounted for 45.9% of deaths from secondhand smoke globally, leading both genders (figure 1A). Within CVD, ischaemic heart disease was the most prevalent, comprising 66.4% of cases (figure 1B). The South-East Asia Region had the highest number of CVD deaths due to secondhand smoke among WHO regions, with a significant 100.7% increase to 153.0 thousand deaths in 2019 (online supplemental table 1 and figure 2). From 1990 to 2019, the Western Pacific Region saw the fastest increase in all-age CVD mortality, surging by 33.1% to 11.4 per 100 000 population (figure 2B).

In 2019, the global age-standardised death rate for CVD deaths linked to secondhand smoke was 7.4 cases per 100 000 population (95% UI: 6.1 to 8.9) (online supplemental table 1). The Eastern Mediterranean Region had the highest rate, with 15.3 cases per 100 000 (online supplemental table 1 and figure 2A). Globally, the age-standardised death rate declined by 35.1% (online supplemental table 1). Disparities in global age-standardised and all-age mortality rates, evident across genders and CVD subtypes (table 1), highlight the importance of considering age composition in CVD-related mortality assessments (table 2).

**Table 2:** The global trends in the CVD burden attributable to secondhand smoke by sex and CVD subtype from 1990 to 2019

Subtype	Deaths		All-age mortality		Age-standardised mortality		Net drift of mortality (APC model estimates),† % per year
	Number in 2019,* n	Change of numbers 1990–2019, %	Rate in 2019, per 100 000	Percent change 1990–2019, %	Rate in 2019, per 100 000	Percent change 1990–2019, %	
CVD							
Both	598 474.7 (489 749.3, 713 482.2)	38.4 (26.8, 49.5)	7.73 (6.33, 9.22)	−4.3 (–12.3, 3.4)	7.43 (6.09, 8.85)	−35.1 (–40.3, –30.0)	−1.65 (–1.69, –1.60)
Female	320 041.7 (261 416.7, 384 510.5)	32.8 (16.7, 48.2)	8.30 (6.78, 9.97)	−8.6 (–19.7, 2.1)	7.33 (6.00, 8.80)	−37.0 (–44.4, –30.0)	−1.78 (–1.83, –1.73)
Male	278 433.1 (224 397.9, 337 392.9)	45.4 (30.6, 61.9)	7.17 (5.78, 8.69)	0.9 (–9.3, 12.3)	7.50 (6.07, 9.10)	−32.6 (–38.9, –25.4)	−1.47 (–1.54, –1.41)
Ischaemic heart disease							
Both	397 420.0 (319 896.5, 477 621.8)	47.2 (34.9, 58.6)	5.14 (4.13, 6.17)	1.8 (–6.7, 9.7)	4.94 (3.98, 5.93)	−31.1 (–36.3, –26.2)	−1.40 (–1.45, –1.34)
Female	203 847.8 (164 262.2, 248 090.1)	46.9 (31.0, 63.1)	5.29 (4.26, 6.43)	1.2 (–9.8, 12.3)	4.67 (3.77, 5.68)	−30.7 (–38.2, –23.6)	−1.41 (–1.47, –1.35)
Male	193 572.2 (153 575.9, 235 381.1)	47.5 (33.9, 62.3)	4.99 (3.96, 6.06)	2.4 (–7.1, 12.6)	5.20 (4.12, 6.36)	−31.4 (–37.5, –25.1)	−1.38 (–1.46, –1.31)
Ischaemic stroke							
Both	86 232.5 (63 681.1, 112 648.2)	51.5 (33.3, 71.1)	1.11 (0.82, 1.46)	4.8 (–7.8, 18.3)	1.10 (0.81, 1.43)	−32.6 (–40.5, –24.2)	−1.67 (–1.71, –1.62)
Female	49 998.5 (37 049.8, 65 596.6)	39.8 (20.0, 63.3)	1.30 (0.96, 1.70)	−3.7 (–17.4, 12.5)	1.14 (0.84, 1.49)	−36.0 (–44.1, –25.9)	−1.95 (–2.01, –1.89)
Male	36 234.0 (25 555.9, 47 785.9)	71.4 (43.1, 98.6)	0.93 (0.66, 1.23)	18.9 (–0.7, 37.9)	1.03 (0.74, 1.36)	−26.4 (–37.6, –15.6)	−1.25 (–1.31, –1.18)
Stroke							
Both	201 054.8 (148 762.7, 257 594.3)	23.7 (9.7, 38.7)	2.60 (1.92, 3.33)	−14.5 (–24.1, –4.1)	2.49 (1.86, 3.18)	−41.8 (–48.2, –34.9)	−2.08 (–2.12, –2.04)
Female	116 193.8 (87 005.0, 149 700.2)	13.6 (2.0, 32.0)	3.01 (2.26, 3.88)	−21.8 (–32.5, –9.1)	2.66 (2.00, 3.42)	−45.7 (–53.0, –36.8)	−2.34 (–2.39, –2.29)
Male	84 860.9 (60 473.5, 111 982.7)	40.8 (20.5, 64.2)	2.19 (1.56, 2.89)	−2.3 (–16.4, 14.0)	2.30 (1.65, 3.01)	−35.1 (–44.0, –24.7)	−1.66 (–1.72, –1.60)

The all-age mortality is equivalent to the crude mortality rate.

* The parentheses accompanying all GBD health estimates signify 95% uncertainty intervals,; whereas, those for net drift denote 95% CIsconfidence intervals.

† The net drifts, derived from the age-period-cohort model, represent estimates that indicate the overall annual percentage change in mortality, incorporating the influences of calendar time and consecutive birth cohorts.

APCage-period-cohortCVD, cardiovascular disease

### Trends in the cardiovascular disease burden attributable to secondhand smoke across socio-demographic index quintiles

**Figure 3:**
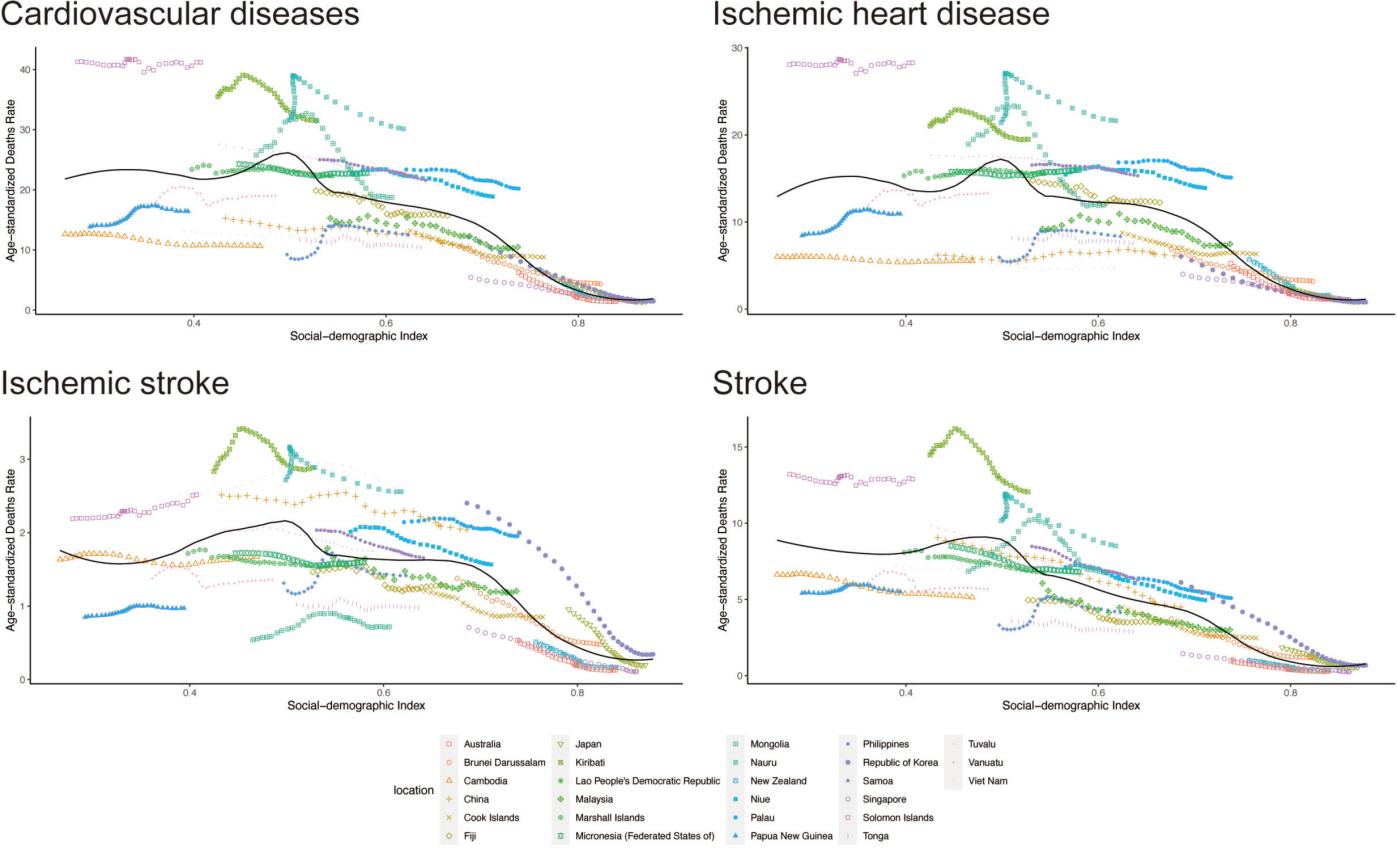
The correlation between SDI levels and the age-standardised death rate due to CVD caused by secondhand smoke in 27 countries from 1990 to 2019. CVD, cardiovascular disease; SDI, Sociodemographic Index.

Online supplemental table 1 outlines global and 27-country age-standardised CVD death rates from secondhand smoke in 2019. Across SDI levels, notable shifts in mortality rates were observed, with higher SDI nations tending to have lower CVD-related mortality rates (figure 3). This consistent trend applies to all subtypes of CVD and both genders (figure 3 and online supplemental figure S2).

It is noteworthy that the age-standardised mortality rates of cardiovascular diseases caused by secondhand smoke have been decreasing in almost all regions and countries, with the exception of Philippines and Papua New Guinea (online supplemental table 1). Among them, the percentage change in age-standardised mortality rate in South Korea reached an astonishing −87.8% ranking first in the world.

### Temporal trends in cardiovascular disease burden attributable to secondhand smoke across age groups

**Figure 4:**
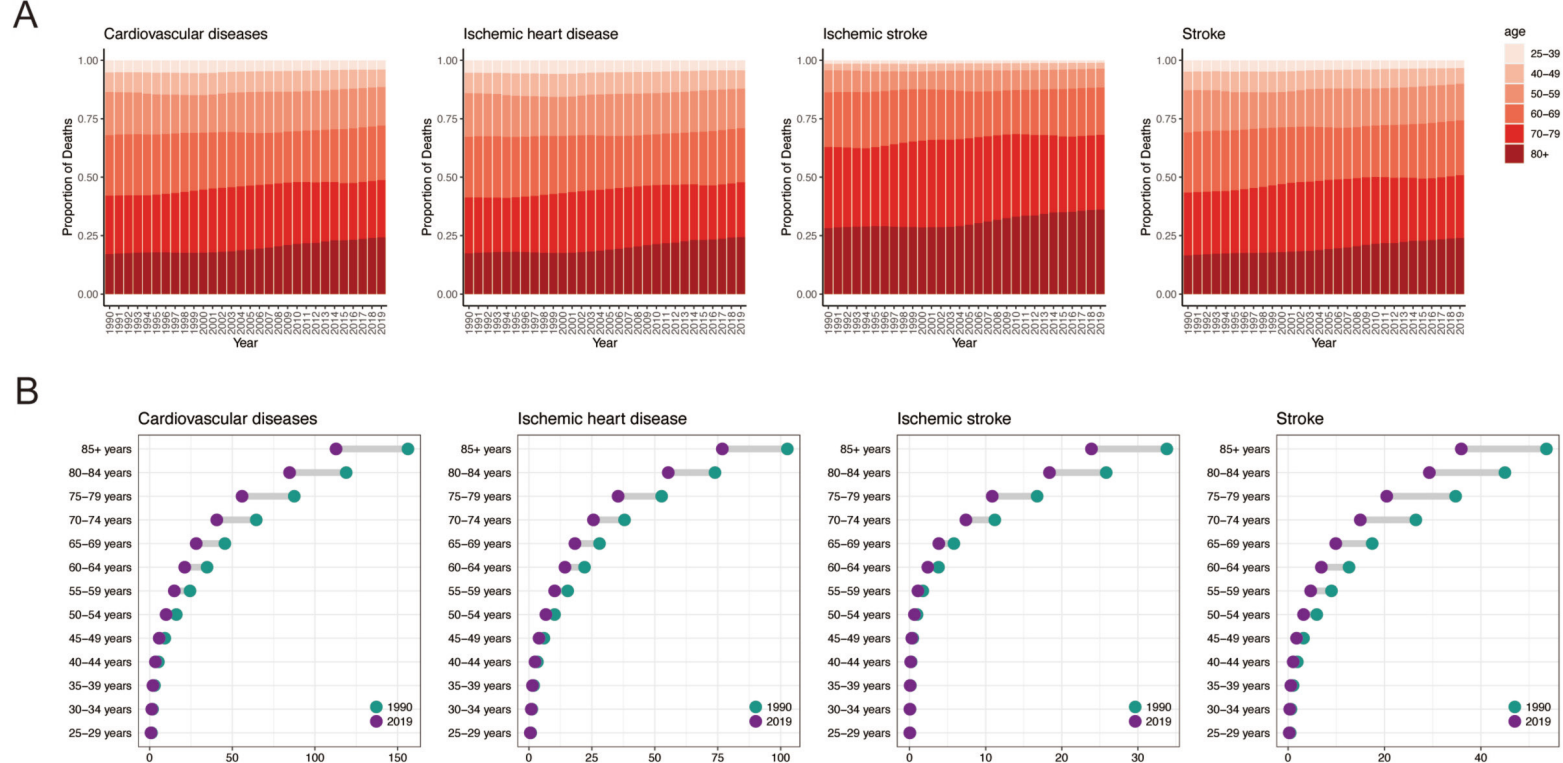
The global temporal variation in the mortality rate attributed to CVD-related mortality caused by secondhand smoke across age groups from 1990 to 2019. (**A**) The relative proportion of CVD-related mortality. (**B**) The temporal changes in the mortality rate of CVD. CVD, cardiovascular disease.

Over the past three decades, a gradual shift towards older age cohorts (aged 80 and above) has been observed in the population exposed to secondhand smoke, accompanied by a change in CVD-related mortality patterns across all subtypes (figure 4A). This trend is consistent across most countries and applies to ischaemic heart disease, ischaemic stroke, and stroke (online supplemental figures S3−S6). Notably, females exposed to secondhand smoke tend to experience CVD-related mortality at older ages (over 80); whereas, males are more susceptible during middle age (40–79 years) (online supplemental figure S7).

Between 1990 and 2019, the CVD mortality rate declined among populations exposed to secondhand smoke, regardless of sex, with a more significant reduction observed in older age groups. During this period, mortality rates for specific CVD types varied, with stroke experiencing the most notable improvement, a remarkable −32.9% decrease (figure 4B). However, an increase in CVD-related mortality was observed in Cambodia, Kiribati, Laos, Micronesia, Papua New Guinea and the Solomon Islands (online supplemental figure S8). Additionally, country-specific variations were noted in deaths from ischaemic heart disease (online supplemental figure S9), ischaemic stroke (online supplemental figure S10), and overall stroke (online supplemental figure S11).

When considering sex differences, males aged 85 and above displayed smaller reductions in CVD (−24.4%), ischaemic stroke (−22.4%), and stroke (−25.1%) death rates compared with their female counterparts (online supplemental figure S12). In contrast, females experienced more substantial reductions, specifically (−29%). However, the disparity in the degree of reduction between males and females was less significant and less distinct when it came to ischaemic heart disease (online supplemental figure S12).

### The local drift, age, period, and cohort effects on cardiovascular disease burden attributable to secondhand smoke

**Figure 5:**
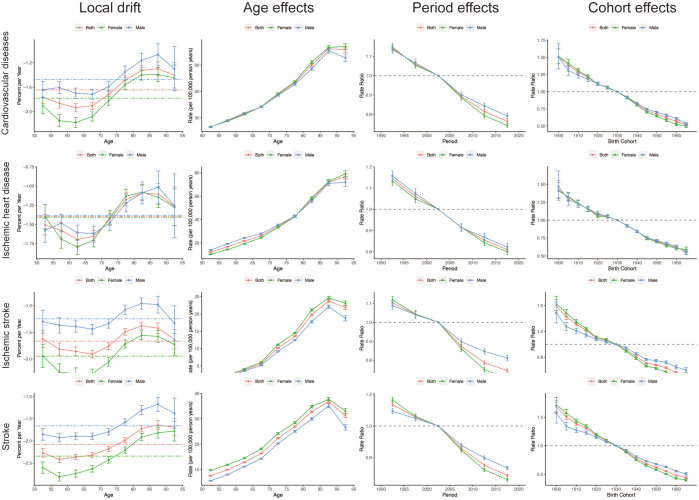
The global local drifts, age effects, period effects and cohort effects in CVD-related mortality attributed to secondhand smoke from 1990 to 2019. CVD, cardiovascular disease.

Figure 5 offers profound insights into the measures derived from the APC model, encompassing local drift, as well as age, period and cohort effects. Specifically, age effects illustrate the longitudinal age curve. Period effects showcase the relative risk of mortality across various time periods. Cohort effects highlight the relative risk of mortality among different cohorts.

Based on the APC model, the annual net drift in CVD mortality is −1.65%. Among WHO regions, the Americas exhibit the most significant decline at −3.65%, while Africa shows the smallest change at −0.98% (figure 5, online supplemental tables 1 and 2). Across age groups and genders, the local drift consistently declines (figure 5, online supplemental table 2). However, gender disparities exist, with males experiencing a marginally smaller decline in CVD-related mortality (−1.47%) than females (−1.78%). Similar trends are observed for ischaemic heart disease and ischaemic stroke, with females showing greater declines than males (figure 5, online supplemental table 2).

The longitudinal analysis of CVD-related mortality by sex demonstrated a gradual escalation in mortality rates as age increased in populations exposed to secondhand smoke. Despite a comparable pattern of mortality rise in both genders across all three categories, there was a discernible shift in the rate of increase observed among individuals within the 75–80-year age bracket (figure 5 and online supplemental table 2). In terms of period effects, populations exposed to secondhand smoke exhibited comparable patterns of reduced CVD-related mortality across all causes.

Notably, the observed gender differences in CVD-related mortality decline highlight the need for tailored interventions to address these disparities. For instance, the slightly smaller decline in CVD-related mortality among males compared with females suggests that males may require more targeted prevention and treatment strategies. Further research is needed to understand the underlying factors contributing to these gender differences, such as differences in smoking behaviours, exposure to secondhand smoke, and physiological responses to smoke exposure.

### The age–period–cohort effects on cardiovascular disease burden attributable to secondhand smoke for exemplar countries

It is important to mention that the variations observed in local drift, age, period and cohort effects among different countries across the world can largely be explained by variations in data quality and the comparatively small size of their total populations. Consequently, some nations that comprise national groups with limited epidemiological studies tend to be under-represented.

Among the 27 countries studied, only three exhibited statistically positive net drift (online supplemental table 1). The top five countries with the highest reduction rates in statistically negative net drift were the Republic of Korea (−8.02%), Australia (−5.86%), Singapore (−5.18%), New Zealand (−4.89%) and Japan (−4.30%) (online supplemental table 1 and figure S3). Notably, all five belong to the high SDI category and have achieved substantial reductions over the past 30 years, marked by a significant shift in CVD-related mortality to older demographics.

Encouraging trends in period and birth cohort patterns have been observed (online supplemental figures S13 and S14). Notably, Japan experienced the most significant change, with the lowest CVD-related death rate among individuals under 80 in 2019 (50.8%), a 27.5% decrease from 1990 (online supplemental figure S3). This age effect demonstrates a gradual transition (online supplemental figure S15). In contrast, the Philippines, a low-middle-SDI country, uniquely shifted its mortality distribution towards individuals under 80 (online supplemental figures S3 and S16).

In 2019, 85.3% of CVD deaths in under 80-year-olds were linked to secondhand smoke, with risk escalating in recent birth cohorts (online supplemental figure S14). This pattern is also evident in low-SDI and low-middle-SDI countries, highlighting prevention and primary care gaps in lower-SDI nations.

### Long-term trends in cardiovascular disease–related mortality among populations with secondhand smoke from 1990 to 2019

**Figure 6:**
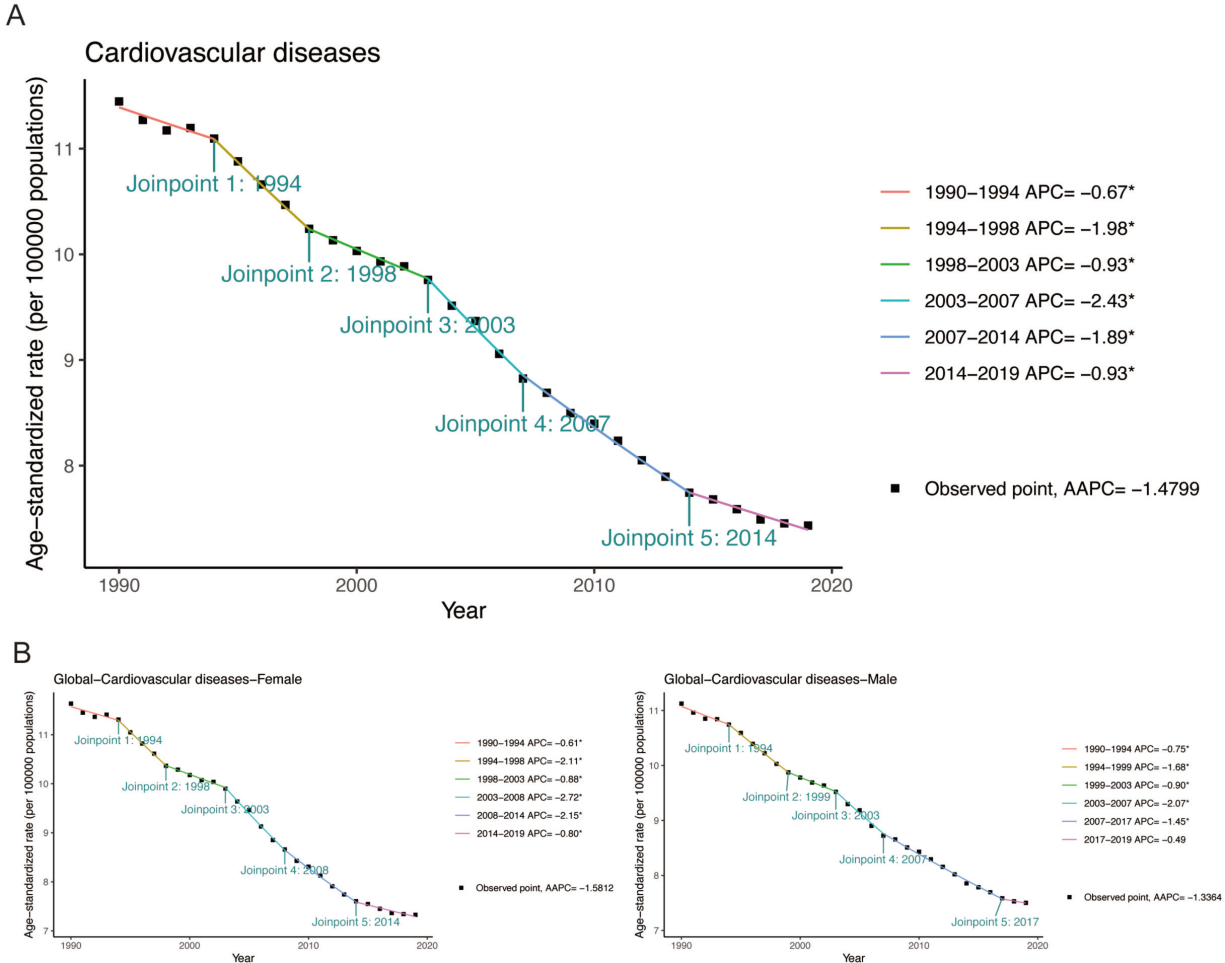
Joinpoint regression analysis of the worldwide age-standardised death rate attributed to CVD caused by secondhand smoke from 1990 to 2019. (**A**) Joinpoint regression analysis of the globally age-standardised death rate for both sexes attributed to CVD caused by secondhand smoke from 1990 to 2019. (**B**) Joinpoint regression analysis of the globally sex-specific age-standardised death rate attributed to CVD caused by secondhand smoke from 1990 to 2019. CVD, cardiovascular disease.

Figure 6 depicts 30-year trends in CVD mortality from secondhand smoke, showing a global decline. The global incidence of CVD-related mortality was divided into six phases, with a notable 2.43% reduction in mortality during 2003–2007. The overall AAPC for this period was −1.48%. Notably, females exhibited a higher AAPC in CVD-related mortality than males over 30 years (online supplemental figure S17 and online supplemental table 3). Among the six WHO regions, the USA experienced the most pronounced decline in AAPC for secondhand smoke-related CVD mortality, at 3.2%. From 1990 to 2019, US mortality trends were divided into six phases, with the greatest decline occurring in 2002–2006 (−5.02%), the highest among all regions and periods (online supplemental figure S17 and online supplemental table 3). Detailed joinpoint regression results for six regions and 27 countries are provided in online supplemental figure S17 and online supplemental table 3.

## Discussion

In our research, we acknowledge the GBD’s updated data but recognise COVID-19’s unprecedented impact altering CVD burden, disrupting trend estimates of secondhand smoke exposure. Consequently, GBD 2021 data lacks representativeness for actual burden. Our goal is to estimate disease burden under normalised conditions, aiding health policy formulation. Thus, we excluded this data to provide accurate, pandemic-unbiased insights on secondhand smoke’s impact on cardiovascular health.

### Trends in the global burden of CVD caused by secondhand smoke

Globally, CVD represents a significant concern for public health.^21^ Secondhand smoke, as one of the factors affecting cardiovascular disease,^22^ is increasingly receiving more attention because it is a risk factor that can be improved through efforts and is considered an effective measure for the prevention and control of non-communicable diseases.^23^ Our study reveals alarming trends in CVD deaths from secondhand smoke, both in numbers and percentages. To our knowledge, it is the first longitudinal analysis using APC and joinpoint regression on GBD 2019 data, showing country, sex and CVD subtype variations (see graphical abstract). These findings inform effective public health interventions and policies globally.

Over three decades, global age-standardised CVD mortality and net drift declined, yet total CVD deaths from secondhand smoke soared 38.4% to 598 474.7 cases (online supplemental table 1). Globally, 3.1% of CVD deaths in 2019 were attributed to secondhand smoke, underscoring its importance. Urgent interventions are crucial to mitigate its effects on CVD mortality. A shift to older populations exposed to secondhand smoke suggests improved survival. In analysing temporal trends among various age groups, the study noted a progressive transition from secondhand smoke exposure to older populations worldwide, suggesting an enhancement in survival rates for this demographic over time. Although figure 4 indicates an overall positive trend, it is crucial to pay particular attention to specific countries, such as the Philippines, Papua New Guinea and Vanuatu mentioned earlier, that do not conform to this pattern. These countries may necessitate tailored healthcare interventions and preventative strategies.

In 2020, the global population aged 65 and above was 727 million, accounting for about 9.3% of the total population. It is expected to double to 1.5 billion (16%) by 2050.^3^ The significance of taking into account the age distribution of CVD-related mortality becomes evident when interpreting mortality data and developing interventional strategies. The urgency of taking action to tackle population ageing has become increasingly apparent.^24^ Taking necessary actions throughout the entire lifecycle to reduce secondhand smoke exposure, along with targeted protective measures for specific age groups^25^ and an increase in screening and educational interventions,^26^ may be of significant importance in alleviating the global burden of cardiovascular diseases.

### National and gender differences in CVD caused by secondhand smoke

Our research explores global disparities in CVD burden from secondhand smoke, correlating SDI levels. The SDI serves as a quantitative indicator of a country’s socioeconomic status, encompassing the total fertility rate among individuals under 25, the average education level among those aged 15 and above, and the per capita income distribution.^27^ The overall progress in sociodemographic factors holds significant influence in shaping health outcomes, extending beyond mere healthcare expenditure, accessibility and quality.^28^ Study outcomes reveal that countries with higher SDI exhibit lower age-standardised mortality rates; whereas, those with lower SDI consistently show higher rates, regardless of gender (figure 3). Among 27 studied countries, 17 have middle/lower SDI, notably including 14 Pacific Islands with shared political/social backgrounds, facing challenges from non-communicable diseases in their health systems and economies.^29^ Limited sample size and data quality may compromise the APC model’s accuracy in estimating CVD burden trends among secondhand smoke-exposed populations, requiring cautious interpretation. In 17 middle/lower SDI countries, age-standardised mortality rates surpassed global averages, trending unfavourably upwards (Supplementary Table 1). The WHO aims to reduce premature mortality from non-communicable diseases by 25%, emphasising the significant impact on adult deaths.^30^ In lower socioeconomic development contexts, inadequate accessibility and quality of healthcare services are observed.^31^ Comprehensive epidemiological studies are essential for identifying public health priorities and reducing CVD burden. While progress in secondhand smoke control is seen in higher SDI countries, lower SDI nations urgently need action and investment in prevention to enhance health access. In this study, the APC model is instrumental in analysing the temporal changes in the mortality rate of CVD related to secondhand smoke, identifying causal factors, and assisting us in determining targeted prevention and treatment measures.

Globally, local variations in mortality rates consistently declined, suggesting improved CVD-related mortality from secondhand smoke, with a notable sex disparity: females generally experienced greater reductions (figure 5). Statistical data indicate that women are more affected by secondhand smoke than men, with proportions of 35% for women and 33% for men.^32^ The potential genetic and molecular mechanisms underlying the adverse effects of secondhand smoke on both sexes remain to be fully elucidated.^33^ Joinpoint regression analysis showed a more pronounced decline in male mortality rates (−1.34% AAPC) than females (−1.58% AAPC), globally (figure 6). Furthermore, discernible disparities in the prevalence of CVD were observed across various countries and regions (online supplemental figure S17). This may be attributed to the significant differences in secondhand smoke exposure among countries, stemming from their varying cultural backgrounds and tobacco control policies.^34^ Understanding current CVD epidemiological characteristics is crucial for developing effective, sustainable prevention and control measures.

### Limitation

The present study has limitations that necessitate cautious interpretation for public health policy. Specifically, GBD’s mutually exclusive approach to assigning a single CVD cause per mortality case may be restrictive, especially in elderly individuals where mortality often involves multiple factors.^35^ First, the GBD study adopts a mutually exclusive approach in assigning causes of death, attributing a single CVD cause to each mortality case.^35^ However, in elderly individuals, mortality often stems from a multifaceted interplay of factors, particularly in the presence of multiple comorbidities, rendering it improbable for CVD to be designated as the primary cause of death. Improving data collection, case definitions and disease registries would enhance cross-country comparisons and disease burden management.^36^ Second, limited or suboptimal raw data in low-income and middle-income countries, particularly those with smaller populations, introduces substantial uncertainty in GBD mortality and time trend estimates derived from APC models. Standardising and strengthening epidemiological investigations of cardiovascular diseases in these areas may help to improve the situation. Furthermore, although this study focuses on individuals affected by secondhand smoke, due to data deficiencies in the 2019 GBD, there is a lack of information on individual-specific dose, type and duration of exposure to secondhand smoke. Lastly, the burden of secondhand smoke on CVD was assessed at the national and regional levels, without considering specific intra-regional variations, such as differences between tobacco-producing and non-producing areas, urban and rural areas, plains and plateaus. Further research may facilitate the discovery of novel variations.

## Conclusion

This comprehensive study tracks 30-year trends in CVD mortality from secondhand smoke across 6 WHO regions and 27 countries. Findings emphasise the modifiable risk of secondhand smoke for CVDs, guiding interventions to reduce global CVD mortality. Amid global ageing, exploring factors underpinning regional, gender and CVD subtype variations within countries is vital for targeted public health policies.

## supplementary material

10.1136/openhrt-2024-003079 online supplemental file 1

## Data Availability

Data are available in a public, open access repository.

## References

[1] Rajagopalan S, Landrigan PJ (2021). Pollution and the Heart. N Engl J Med.

[2] GBD 2019 Diseases and Injuries Collaborators (2020). Global burden of 369 diseases and injuries in 204 countries and territories, 1990-2019: a systematic analysis for the Global Burden of Disease Study 2019. Lancet.

[3] Department of Economic and Social Affairs Population Division (2022). World population prospects 2022.

[4] Carreras G, Lugo A, Gallus S (2019). Burden of disease attributable to second-hand smoke exposure: a systematic review. Prev Med.

[5] WHO (2023). Seven out of 10 people protected by at least one tobacco control measure.

[6] Institute for Health Metrics and Evaluation (2019). Global burden of disease study 2019 (GBD 2019) data resources.

[7] Murray CJ, Ezzati M, Lopez AD (2003). Comparative quantification of health risks conceptual framework and methodological issues. Popul Health Metr.

[8] Institute for Health Metrics and Evaluation (2020). Global burden of disease 2019 risk factor factsheets.

[9] Institute for Health Metrics and Evaluation (2020). Global burden of disease 2019 disease, injury, and impairment factsheets.

[10] GBD 2019 Demographics Collaborators (2020). Global age-sex-specific fertility, mortality, healthy life expectancy (HALE), and population estimates in 204 countries and territories, 1950-2019: a comprehensive demographic analysis for the Global Burden of Disease Study 2019. Lancet.

[11] Institute for Health Metrics and Evaluation (2020). Secondhand smoke—level 3 risk.

[12] Institute for Health Metrics and Evaluation (2020). Cardiovascular diseases—level 2 cause.

[13] Murray CJ, Lopez AD (1997). Global mortality, disability, and the contribution of risk factors: Global Burden of Disease Study. Lancet.

[14] GBD 2017 Causes of Death Collaborators (2018). Global, regional, and national age-sex-specific mortality for 282 causes of death in 195 countries and territories, 1980-2017: a systematic analysis for the Global Burden of Disease Study 2017. Lancet.

[15] Rosenberg PS, Anderson WF (2011). Age-period-cohort models in cancer surveillance research: ready for prime time?. Cancer Epidemiol Biomarkers Prev.

[16] Falcaro M, Castañon A, Ndlela B (2021). The effects of the national HPV vaccination programme in England, UK, on cervical cancer and grade 3 cervical intraepithelial neoplasia incidence: a register-based observational study. Lancet.

[17] Zumsteg ZS, Luu M, Rosenberg PS (2023). Global epidemiologic patterns of oropharyngeal cancer incidence trends. J Natl Cancer Inst.

[18] Rosenberg PS, Check DP, Anderson WF (2014). A web tool for age-period-cohort analysis of cancer incidence and mortality rates. Cancer Epidemiol Biomarkers Prev.

[19] Sun P, Wen H, Liu X (2022). Time trends in type 2 diabetes mellitus incidence across the BRICS from 1990 to 2019: an age-period-cohort analysis. BMC Public Health.

[20] Zhang J, Ma B, Han X (2022). Global, regional, and national burdens of HIV and other sexually transmitted infections in adolescents and young adults aged 10-24 years from 1990 to 2019: a trend analysis based on the Global Burden of Disease Study 2019. Lancet Child Adolesc Health.

[21] Greenberg H (2013). Global cardiovascular disease and the academic public health curriculum. Prog Cardiovasc Dis.

[22] Zhu BQ, Sun YP, Sievers RE (1994). Exposure to environmental tobacco smoke increases myocardial infarct size in rats. Circulation.

[23] Zusman M, Gassett AJ, Kirwa K (2021). Modeling residential indoor concentrations of PM(2.5), NO(2), NO(x), and secondhand smoke in the Subpopulations and Intermediate Outcome Measures in COPD (SPIROMICS) Air study. Indoor Air.

[24] Kasai DT (2021). Preparing for population ageing in the Western Pacific Region. *Lancet Reg Health West Pac*.

[25] Meeker JD, Missmer SA, Vitonis AF (2007). Risk of spontaneous abortion in women with childhood exposure to parental cigarette smoke. Am J Epidemiol.

[26] Middleton C, Bruns D (2019). Improving Screening and Education for Secondhand Smoke Exposure in Primary Care Settings. Am J Nurs.

[27] Liang Y, Zhang N, Wang M (2023). Distributions and Trends of the Global Burden of Colorectal Cancer Attributable to Dietary Risk Factors over the Past 30 Years. Nutrients.

[28] Molassiotis A, Kwok SWH, Leung AYM (2022). Associations between sociodemographic factors, health spending, disease burden, and life expectancy of older adults (70 + years old) in 22 countries in the Western Pacific Region, 1995-2019: estimates from the Global Burden of Disease (GBD) Study 2019. Geroscience.

[29] Tuitama LT, Young-Soo S, Clark H (2014). Acting on the Pacific crisis in non-communicable diseases. Lancet.

[30] Stringhini S, Carmeli C, Jokela M (2017). Socioeconomic status and the 25 × 25 risk factors as determinants of premature mortality: a multicohort study and meta-analysis of 1·7 million men and women. Lancet.

[31] Robertson C, Ecob R (1999). Simultaneous modelling of time trends and regional variation in mortality rates. Int J Epidemiol.

[32] Oberg M, Jaakkola MS, Woodward A (2011). Worldwide burden of disease from exposure to second-hand smoke: a retrospective analysis of data from 192 countries. Lancet.

[33] Schmid P, Karanikas G, Kritz H (1996). Passive smoking and platelet thromboxane. Thromb Res.

[34] Ezzati M, Lopez AD, Rodgers A (2002). Selected major risk factors and global and regional burden of disease. Lancet.

[35] Roth GA, Mensah GA, Johnson CO (2020). Global Burden of Cardiovascular Diseases and Risk Factors, 1990-2019: Update From the GBD 2019 Study. J Am Coll Cardiol.

[36] Su Z, Zou Z, Hay SI (2022). Global, regional, and national time trends in mortality for congenital heart disease, 1990-2019: An age-period-cohort analysis for the Global Burden of Disease 2019 study. EClinicalMedicine.

