# Supplementary material for: Global, regional and national burdens of cardiovascular disease attributable to secondhand smoke from 1990–2019: an age-period-cohort analysis
Source: Open Heart. 2025 Feb 11;12(1):e003079. doi: 10.1136/openhrt-2024-003079 (PMC11815441; doi:10.1136/openhrt-2024-003079)
Supplement: online supplemental file 1 [file openhrt-12-1-s001.docx]

**Supplemental materials**

**Supplementary Table 1.** The trends in the CVD burden attributable to secondhand smoke in 6 regions and 27 countries from 1990 to 2019

**Supplementary Table 2.** The local drift of CVD deaths attributable to secondhand smoke globally and in 27 countries from 1990 to 2019.

**Supplementary Table 3.** The results of the Joinpoint regression analysis, showing the trends in age-standardized death rates (per 100,000 persons) for CVD attributable to secondhand smoke among both sexes, females and males in the world and six main regions from 1990 to 2019.

**Supplementary Figure 1.** The percentage of death number for CVD, ischemic heart disease, ischemic stroke and stroke in five groups (0-49, 50-54, 55-89, 90-94, >95 years) attributable to secondhand smoke for both sexes combined globally from 1990 to 2019.

**Supplementary Figure 2.** The correlation of SDI levels with the age-standardized death rate for CVD caused by secondhand smoke in 27 countries from 1990 to 2019.

**Supplementary Figure 3.** The temporal variation in the relative proportion of CVD deaths attributed to secondhand smoke across various age groups globally and in 27 countries and globally from 1990 to 2019.

**Supplementary Figure 4.** The temporal variation in the relative proportion of ischemic heart disease deaths attributed to secondhand smoke across various age groups globally and in 27 countries from 1990 to 2019.

**Supplementary Figure 5.** The temporal variation in the relative proportion of ischemic stroke deaths attributed to secondhand smoke across various age groups globally and in 27 countries from 1990 to 2019.

**Supplementary Figure 6.** The temporal variation in the relative proportion of stroke deaths attributed to secondhand smoke across various age groups globally and in 27 countries from 1990 to 2019.

**Supplementary Figure 7.** The global temporal variation in the sex-specific relative proportion of CVD deaths attributed to secondhand smoke across various age groups from 1990 to 2019.

**Supplementary Figure 8.** The temporal variation in the death rate for CVD caused by secondhand smoke across age groups globally and in 27 countries between 1990 and 2019.

The dumbbell plot's horizontal axis depicts "Deaths Rates, per 100,000."

**Supplementary Figure 9.** The temporal variation in the death rate for ischemic heart disease caused by secondhand smoke across age groups globally and in 27 countries between 1990 and 2019.

**Supplementary Figure 10.** The temporal variation in the death rate for ischemic stroke caused by secondhand smoke across age groups globally and in 27 countries between 1990 and 2019.

**Supplementary Figure 11.** The temporal variation in the death rate for stroke caused by secondhand smoke across age groups globally and in 27 countries between 1990 and 2019.

**Supplementary Figure 12.** The temporal variation in the sex-specific deaths rate for CVD caused by secondhand smoke across age groups globally and in 27 countries between 1990 and 2019.

**Supplementary Figure 13.** The local drifts of CVD deaths attributable to secondhand smoke globally and in 27 countries from 1990 to 2019.

**Supplementary Figure 14.** The age effects of CVD deaths attributable to secondhand smoke globally and in 27 countries from 1990 to 2019.

**Supplementary Figure 15.** The period effects of CVD deaths attributable to secondhand smoke globally and in 27 countries from 1990 to 2019.

**Supplementary Figure 16.** Thecohort effects of CVD deaths attributable to secondhand smoke globally and in 27 countries from 1990 to 2019.

**Supplementary Figure 17.** The Joinpoint regression analysis of the age-standardized death rate for CVD attributable to secondhand smoke globally and in 27 countries from 1990 to 2019.

**Supplementary Table 1.** The trends in the CVD burden attributable to secondhand smoke in 6 regions and 27 countries from 1990 to 2019

| **Location** | **Deaths** | | **All-age mortality** | | **Age-standardized mortality** | | Net drift of mortality (APC model estimates)†, % per year |
| --- | --- | --- | --- | --- | --- | --- | --- |
|  | **Number in 2019*, n** | **Change of numbers 1990–2019, %** | **Rate in 2019, per 100000** | **Percent change 1990–2019, %** | **Rate in 2019, per 100000** | **Percent change 1990–2019, %** |  |
| Global | 598474.7 (489749.3, 713482.2) | 38.4 (26.8, 49.5) | 7.73 (6.33, 9.22) | -4.3 (-12.3, 3.4) | 7.43 (6.09, 8.85) | -35.1 (-40.3, -30.0) | -1.65 (-1.69, -1.60) |
| **Regions** | | | | | | | |
| African Region | 22343.8 (17332.4, 27713.7) | 63.3 (41.0, 87.2) | 2.03 (1.58, 2.52) | -24.4 (-34.7, -13.3) | 4.99 (3.89, 6.19) | -23.6 (-33.6, -13.1) | -0.98 (-1.09, -0.86) |
| Eastern Mediterranean Region | 61899.2 (49456.2, 74429.6) | 77.1 (55.2, 102.2) | 8.53 (6.82, 10.26) | -7.7 (-19.1, 5.4) | 15.25 (12.27, 18.30) | -23.7 (-32.5, -13.8) | -1.09 (-1.15, -1.03) |
| European Region | 99019.8 (82081.4, 116606.0) | -19.9 (-25.7, -14.1) | 10.63 (8.81, 12.52) | -26.0 (-31.4, -20.6) | 6.13 (5.11, 7.21) | -47.4 (-51.1, -43.9) | -2.69 (-2.81, -2.57) |
| Region of the Americas | 39890.7 (32471.4, 47426.1) | -18.4 (-23.4, -12.9) | 3.95 (3.21, 4.69) | -42.2 (-45.7, -38.3) | 3.15 (2.56, 3.75) | -61.2 (-63.5, -58.6) | -3.65 (-3.70, -3.60) |
| South-East Asia Region | 152973.2 (122153.9, 185582.7) | 100.7 (74.3, 127.3) | 7.59 (6.06, 9.21) | 30.2 (13.1, 47.5) | 9.28 (7.40, 11.26) | -21.9 (-32.0, -11.5) | -1.05 (-1.22, -0.88) |
| Western Pacific Region | 221036.3 (174180.4, 269542.9) | 64.6 (36.9, 97.3) | 11.43 (9.01, 13.94) | 33.1 (10.7, 59.5) | 8.43 (6.68, 10.29) | -33.7 (-44.6, -21.4) | -1.31 (-1.38, -1.24) |
| **Countries** | | | | | | | |
| Australia | 586.8 (471.4, 715.3) | -50.8 (-57.3, -42.9) | 2.39 (1.92, 2.91) | -66.2 (-70.7, -60.8) | 1.43 (1.15, 1.73) | -77.1 (-79.9, -73.8) | -5.86 (-6.22, -5.49) |
| Brunei Darussalam | 12.8 (10.1, 15.9) | 21.7 (0.1, 49.1) | 2.9 (2.3, 3.6) | -28.0 (-40.8, -11.8) | 4.4 (3.5, 5.3) | -58.9 (-65.7, -50.4) | -3.26 (-7.63, -1.33) |
| Cambodia | 1171.0 (894.4, 1474.9) | 114.5 (66.8, 168.3) | 7.05 (5.39, 8.88) | 34.1 (4.3, 67.7) | 10.67 (8.19, 13.31) | -15.6 (-34.4, 3.1) | -0.56 (-1.02, -0.10) |
| China | 189264.9 (147595.5, 232891.6) | 70.1 (37.7, 109.5) | 13.31 (10.38, 16.37) | 41.6 (14.6, 74.4) | 10.55 (8.32, 13.01) | -30.9 (-43.7, -16.3) | -1.22 (-1.30, -1.13) |
| Cook Islands | 2.1 (1.6, 2.7) | 28.4 (0.5, 66.1) | 11.67 (8.92, 14.98) | 35.8 (6.3, 75.7) | 8.80 (6.67, 11.26) | -33.4 (-47.5, -14.2) | -1.52 (-9.51, 7.18) |
| Fiji | 111.8 (81.1, 147.6) | 54.7 (17.1, 106.0) | 12.27 (8.90, 16.20) | 28.9 (-2.5, 71.7) | 15.72 (11.46, 20.47) | -20.8 (-38.7, 3.7) | -0.86 (-2.43, 0.73) |
| Japan | 4965.7 (3844.0, 6148.7) | -29.7 (-40.1, -18.8) | 3.89 (3.01, 4.81) | -30.7 (-41.0, -20.0) | 1.42 (1.14, 1.71) | -67.6 (-70.9, -63.8) | -4.30 (-4.50, -4.11) |
| Kiribati | 23.4 (16.9, 30.7) | 62.6 (23.6, 112.5) | 19.68 (14.28, 25.84) | 1.6 (-22.8, 32.7) | 31.55 (23.31, 40.70) | -11.1 (-31.3, 13.9) | -0.34 (-5.58, 5.19) |
| Lao People's Democratic Republic | 597.4 (447.2, 760.3) | 60.2 (19.8, 112.0) | 8.35 (6.25, 10.62) | -7.1 (-30.5, 22.9) | 15.03 (11.50, 18.87) | -22.7 (-40.7, 0.5) | -1.14 (-1.74, -0.55) |
| Malaysia | 2654.9 (1930.3, 3457.3) | 98.9 (54.4, 150.3) | 8.48 (6.17, 11.05) | 12.2 (-12.9, 41.1) | 10.49 (7.60, 13.51) | -31.4 (-46.1, -14.3) | -1.44 (-1.71, -1.17) |
| Marshall Islands | 7.9 (5.6, 10.9) | 108.4 (58.5, 170.5) | 13.97 (9.84, 19.22) | 67.6 (27.5, 117.6) | 22.29 (16.20, 29.58) | -4.7 (-25.7, 20.8) | -0.41 (-7.45, 7.16) |
| Micronesia (Federated States of) | 16.2 (10.6, 22.8) | 42.6 (-4.4, 96.8) | 15.83 (10.39, 22.35) | 45.6 (-2.4, 101.0) | 22.81 (15.85, 31.33) | -6.1 (-33.1, 27.1) | -0.18 (-4.51, 4.34) |
| Mongolia | 400.6 (293.7, 527.2) | 58.0 (20.0, 111.2) | 11.83 (8.67, 15.56) | 0.5 (-23.7, 34.3) | 18.75 (14.18, 24.25) | -27.3 (-43.1, -5.7) | -1.96 (-2.62, -1.29) |
| Nauru | 1.4 (1.1, 1.9) | 9.3 (-11.1, 34.0) | 13.58 (10.10, 18.06) | 6.3 (-13.6, 30.2) | 30.15 (23.13, 38.81) | -4.5 (-20.5, 14.5) | -0.40 (-16.12, 18.27) |
| New Zealand | 147.3 (117.5, 181.2) | -42.3 (-49.9, -33.2) | 3.28 (2.61, 4.03) | -56.1 (-61.9, -49.2) | 1.94 (1.54, 2.39) | -71.0 (-74.8, -66.9) | -4.89 (-5.65, -4.13) |
| Niue | 0.4 (0.3, 0.5) | -19.0 (-36.3, 2.9) | 24.19 (18.06, 30.91) | 12.8 (-11.3, 43.2) | 18.86 (14.04, 24.18) | -16.4 (-34.3, 5.8) | -0.75 (-14.93, 15.80) |
| Palau | 4.3 (3.1, 5.7) | 86.6 (35.9, 149.7) | 23.75 (17.43, 31.48) | 59.6 (16.2, 113.6) | 20.20 (15.20, 26.69) | -13.6 (-36.3, 14.0) | -0.65 (-8.60, 8.00) |
| Papua New Guinea | 798.7 (543.0, 1122.5) | 214.4 (136.3, 326.3) | 8.10 (5.50, 11.38) | 30.3 (-2.1, 76.6) | 16.42 (11.29, 22.58) | 18.5 (-7.8, 55.2) | 0.72 (-0.14, 1.59) |
| Philippines | 9225.8 (6909.7, 11784.4) | 325.7 (212.8, 433.0) | 8.23 (6.16, 10.51) | 140.2 (76.5, 200.8) | 12.50 (9.57, 15.92) | 35.2 (4.6, 66.8) | 1.60 (1.38, 1.83) |
| Republic of Korea | 1266.5 (999.2, 1574.7) | -60.9 (-66.9, -52.4) | 2.37 (1.87, 2.95) | -67.6 (-72.5, -60.4) | 1.49 (1.18, 1.85) | -87.8 (-89.4, -85.2) | -8.02 (-8.26, -7.78) |
| Samoa | 31.2 (23.2, 40.2) | 47.9 (13.4, 96.6) | 14.74 (10.96, 19.01) | 14.5 (-12.2, 52.2) | 21.59 (16.21, 27.55) | -13.7 (-32.7, 12.2) | -0.63 (-2.92, 1.71) |
| Singapore | 104.1 (82.9, 128.1) | -13.6 (-25.6. -0.3) | 1.84 (1.46, 2.26) | -53.6 (-60.0, -46.4) | 1.33 (1.06, 1.63) | -75.6 (-78.9, -72.0) | -5.18 (-6.26, -4.08) |
| Solomon Islands | 139.1 (103.0, 184.4) | 128.8 (69.6, 204.7) | 21.21 (15.70, 28.13) | 18.8 (-11.9, 58.2) | 41.20 (31.49, 52.79) | -0.2 (-23.3, 29.0) | 0.02 (-2.85, 2.97) |
| Tonga | 8.2 (6.1, 10.5) | 32.4 (0.3, 73.0) | 8.01 (5.91, 10.25) | 25.3 (5.1, 63.6) | 10.43 (7.72, 13.37) | -10.8 (-31.5, 15.8) | -0.35 (-4.37, 3.84) |
| Tuvalu | 2.2 (1.6, 3.0) | 21.9 (-8.7, 66.7) | 18.91 (13.81, 25.26) | -3.5 (-27.7, 32.0) | 22.50 (16.51, 29.66) | -18.2 (-38.1, 10.8) | -0.76 (-10.20, 9.67) |
| Vanuatu | 34.0 (23.8, 46.6) | 176.8 (103.5, 287.7) | 11.54 (8.09, 15.81) | 42.3 (4.6, 99.3) | 19.02 (13.55, 25.88) | 5.2 (-20.5, 43.4) | -0.28 (-4.15, 3.75) |
| Viet Nam | 8801.3 (6564.9, 11333.4) | 78.3 (34.2, 134.2) | 9.13 (6.81, 11.76) | 25.7 (-5.4, 65.1) | 10.44 (7.83, 13.45) | -20.4 (-39.5, 2.4) | -0.70 (-0.83, -0.57) |
| The all-age mortality is equivalent to the crude mortality rate.  ⁎ The parentheses accompanying all GBD health estimates signify 95% uncertainty intervals, whereas those for net drift denote 95% confidence intervals.  † The net drifts, derived from the age-period-cohort model, represent estimates that indicate the overall annual percentage change in mortality, incorporating the influences of calendar time and consecutive birth cohorts.  **Abbreviation:** CVD, cardiovascular disease. | | | | | | | |

**Supplementary Table 2.** The local drift of CVD deaths attributable to secondhand smoke globally and in 27 countries from 1990 to 2019.

| **Location** | **Sex** | **CVD** | **Ischemic heart disease** | **Ischemic stroke** | **Stroke** |
| --- | --- | --- | --- | --- | --- |
| Global | Both | -1.65 (-1.69, -1.60) | -1.40 (-1.45, -1.34) | -1.67 (-1.71, -1.62) | -2.08 (-2.12, -2.04) |
|  | Female | -1.78 (-1.83, -1.73) | -1.41 (-1.47, -1.35) | -1.95 (-2.01, -1.89) | -2.34 (-2.39, -2.29) |
|  | Male | -1.47 (-1.54, -1.41) | -1.38 (-1.46, -1.31) | -1.25 (-1.31, -1.18) | -1.66 (-1.72, -1.60) |
| African Region | Both | -0.98 (-1.09, -0.86) | -0.82 (-0.96, -0.68) | -0.55 (-0.78, -0.31) | -1.25 (-1.40, -1.10) |
|  | Female | -0.96 (-1.10, -0.83) | -0.76 (-0.92, -0.60) | -0.61 (-0.92, -0.30) | -1.28 (-1.48, -1.09) |
|  | Male | -0.99 (-1.13, -0.85) | -0.88 (-1.05, -0.70) | -0.49 (-0.87, -0.11) | -1.21 (-1.46, -0.96) |
| Eastern Mediterranean Region | Both | -1.09 (-1.15, -1.03) | -1.00 (-1.07, -0.94) | -0.34 (-0.49, -0.18) | -1.38 (-1.50, -1.27) |
|  | Female | -1.15 (-1.23, -1.08) | -1.08 (-1.17, -0.99) | -0.25 (-0.46, -0.04) | -1.37 (-1.52, -1.22) |
|  | Male | -1.02 (-1.10, -0.94) | -0.92 (-1.02, -0.83) | -0.44 (-0.68, -0.20) | -1.40 (-1.58, -1.22) |
| European Region | Both | -2.69 (-2.81, -2.57) | -2.55 (-2.67, -2.44) | -3.25 (-3.40, -3.11) | -3.07 (-3.21, -2.94) |
|  | Female | -2.81 (-2.95, -2.66) | -2.59 (-2.74, -2.45) | -3.54 (-3.70, -3.39) | -3.32 (-3.47, -3.17) |
|  | Male | -2.62 (-2.73, -2.51) | -2.61 (-2.72, -2.50) | -2.80 (-2.93, -2.67) | -2.65 (-2.77, -2.53) |
| Region of the Americas | Both | -3.65 (-3.70, -3.60) | -3.73 (-3.79, -3.68) | -3.83 (-4.00, -3.67) | -3.40 (-3.50, -3.29) |
|  | Female | -3.61 (-3.69, -3.54) | -3.70 (-3.79, -3.61) | -3.80 (-4.06, -3.54) | -3.37 (-3.52, -3.22) |
|  | Male | -3.74 (-3.81, -3.67) | -3.81 (-3.89, -3.74) | -3.94 (-4.16, -3.71) | -3.49 (-3.64, -3.34) |
| South-East Asia Region- | Both | -1.05 (-1.22, -0.88) | -0.74 (-0.92, -0.56) | -0.98 (-1.13, -0.83) | -1.60 (-1.75, -1.45) |
|  | Female | -1.34 (-1.52, -1.15) | -1.03 (-1.23, -0.83) | -1.11 (-1.26, -0.96) | -1.79 (-1.94, -1.63) |
|  | Male | -0.73 (-0.92, -0.54) | -0.41 (-0.61, -0.21) | -0.86 (-1.04, -0.68) | -1.40 (-1.57, -1.22) |
| Western Pacific Region | Both | -1.31 (-1.38, -1.24) | -0.04 (-0.12, 0.05) | -1.17 (-1.23, -1.08) | -2.50 (-2.58, -2.43) |
|  | Female | -1.76 (-1.85, -1.67) | -0.35 (-0.45, -0.25) | -1.65 (-1.76, -1.53) | -3.02 (-3.13, -2.92) |
|  | Male | -0.66 (-0.78, -0.53) | 0.39 (0.25, 0.53) | -0.48 (-0.59, -0.36) | -1.70 (-1.81, -1.59) |
| Australia | Both | -5.86 (-6.22, -5.49) | -6.04 (-6.44, -5.64) | -6.19 (-7.75, -4.60) | -4.98 (-5.86, -4.10) |
|  | Female | -6.16 (-6.83, -5.48) | -6.57 (-7.34, -5.78) | -5.92 (-8.68, -3.08) | -4.85 (-6.21, -3.46) |
|  | Male | -5.89 (-6.34, -5.43) | -6.01 (-6.50, -5.51) | -6.48 (-8.41, -4.52) | -5.22 (-6.39, -4.04) |
| Brunei Darussalam | Both | -3.26 (-7.63, -1.33) | -2.75 (-8.09, 2.90) | -4.23 (-14.93, 7.81) | -4.35 (-11.84, -3.77) |
|  | Female | -3.81 (-9.14, 1.83) | -3.31 (-9.93, 3.79) | -4.55 (-17.82, 10.85) | -4.67 (-13.49, 5.06) |
|  | Male | -2.46 (-12.62, 8.91) | -2.01 (-14.09, 11.78) | -3.54 (-25.98, 25.69) | -3.68 (-21.37, 18.00) |
| Cambodia | Both | -0.56 (-1.02, -0.10) | -0.22 (-0.88, 0.44) | -0.32 (-1.48, 0.86) | -0.88 (-1.52, -0.23) |
|  | Female | -0.84 (-1.36, -0.32) | -0.51 (-1.28, 0.26) | -0.57 (-1.89, 0.77) | -1.12 (-1.83, -0.41) |
|  | Male | -0.04 (-0.61, -0.34) | -0.27 (-1.06, 1.62) | 0.19 (-2.29, 2.73) | -0.29 (-1.84, 1.29) |
| China | Both | -1.22 (-1.30, -1.13) | 0.34 (0.24, 0.44) | -0.93 (-1.04, -0.83) | -2.59 (-2.69, 2.50) |
|  | Female | -1.71 (-1.82, -1.59) | -0.04 (-0.17, 0.10) | -1.46 (-1.60, -1.33) | -3.15 (-3.27, -3.02) |
|  | Male | -0.47 (-0.61, -0.34) | 0.89 (0.75, 1.03) | -0.16 (-0.29, -0.04) | -1.73 (-1.85, 1.60) |
| Cook Islands | Both | -1.52 (-9.51, 7.18) | -1.21 (-10.88, 9.51) | -1.68 (-24.21, 27.53) | -2.21 (-15.82, 13.60) |
|  | Female | -1.77 (-13.54, 11.61) | -1.42 (-16.00, 15.69) | -1.85 (-32.92, 43.62) | -2.38 (-21.07, 20.75) |
|  | Male | -1.32 (-12.42, 11.20) | -1.03 (-14.27, 14.25) | -1.52 (-31.72, 42.04) | -2.04 (-21.25, 21.86) |
| Fiji | Both | -0.86 (-2.43, 0.73) | -0.64 (-2.47, 1.23) | -0.95 (-5.64, 3.97) | -1.55 (-4.56,1.56) |
|  | Female | -0.94 (-3.05, -1.21) | -0.70 (-3.23, -4.91) | -0.73 (-6.92, 5.87) | -1.48 (-5.25, 2.43) |
|  | Male | -0.72 (-3.13, 1.75) | -0.49 (-3.23, 2.32) | -1.18 (-8.37, 6.57) | -1.66 (-6.73, 3.69) |
| Japan | Both | -4.30 (-4.50, -4.11) | -4.07 (-4.24, -3.90) | -5.85 (-6.25, -5.45) | -4.65 (-4.90, -4.40) |
|  | Female | -5.28 (-5.53, -5.05) | 0.32 (-7.98, 9.37) | -7.05 (-7.72, -6.38) | -5.44 (-5.75, -5.12) |
|  | Male | -3.41 (-3.60, -3.21) | -3.19 (-3.44, -2.94) | -4.88 (-5.40, -4.35) | -3.76 (-4.08, -3.44) |
| Kiribati | Both | -0.34 (-5.58, 5.19) | -0.07 (-6.80, 7.15) | -2.30 (-12.77, 13.96) | -0.72 (-8.91 8.20) |
|  | Female | 0.10 (-1.84, -0.42) | 0.32 (-7.98, 9.37) | 0.14 (-16.87, 20.63) | -0.25 (-11.24, 12.12) |
|  | Male | -0.76 (-9.10, 8.35) | -0.49 (-11.92, 12.42) | -0.60 (-18.21, 20.82) | -1.09 (-12.98, 12.43) |
| Lao People's Democratic Republic | Both | -1.14 (-1.74, -0.55) | -1.03 (-1.80, -0.24) | -0.83 (-2.34, 0.70) | -1.30 (-2.22, -0.38) |
|  | Female | -1.13 (-1.84, -0.42) | -1.01 (-1.95, -0.07) | -0.84 (-2.72, 1.07) | -1.29 (-2.36, -0.21) |
|  | Male | -1.08 (-2.24, 0.10) | -0.98 (-2.48, 0.55) | -0.74 (-3.48, 2.08) | -1.22 (-3.06, 0.66) |
| Malaysia | Both | -1.44 (-1.71, -1.17) | -1.18 (-1.51, -0.85) | -1.02 (-1.81, -0.23) | -2.01 (-2.48, -1.53) |
|  | Female | -1.63 (-1.98, -1.28) | -1.35 (-1.78, -0.91) | -1.07 (-2.06, -0.06) | -2.11 (-2.70, -1.52) |
|  | Male | -1.28 (-1.72, -0.84) | -1.09 (-1.60, -0.57) | -0.99 (-2.30, 0.33) | -1.84 (-2.65, -1.02) |
| Marshall Islands | Both | -0.41 (-7.54, 7.16) | -0.15 (-8.76, 9.28) | -0.57 (-20.28, 24.02) | -0.93 (-12.67, 12.39) |
|  | Female | -0.05 (-9.08, 9.88) | 0.22 (-10.82, 12.63) | -0.16 (-24.92, 32.77) | -0.56 (-15.48, 17.00) |
|  | Male | -0.68 (-12.18, 12.34) | -0.39 (-14.51, 16.07) | -0.96 (-30.95, 42.06) | -1.28 (-19.91, 21.70) |
| Micronesia (Federated States of) | Both | -0.18 (-4.51, 4.34) | 0.13 (-5.22, 5.77) | -0.39 (-12.94, 13.96) | -0.79 (-8.03, 7.02) |
|  | Female | -0.17 (-5.75, 5.75) | 0.14 (-6.76, 7.55) | -0.18 (-16.52, 19.35) | -0.72 (-9.96, 9.46) |
|  | Male | -0.22 (-7.11, 7.18) | 0.11 (-8.41, 9.41) | -0.68 (-19.44, 22.45) | -0.89 (-12.25, 11.94) |
| Mongolia | Both | -1.96 (-2.62, -1.29) | 2.73 (-3.50, -1.95) | 0.89 (-3.25, 5.20) | -0.11 (-1.53, 1.32) |
|  | Female | -2.17 (-3.00, -1.34) | -3.14 (-4.12, -2.15) | 0.67 (-4.30, 5.89) | -0.25 (-1.96, 1.50) |
|  | Male | -1.81 (-2.99, -0.62) | -2.44 (-3.79, -1.08) | 1.08 (-6.44, 9.20) | -0.02 (-2.58, 2.61) |
| Nauru | Both | -0.40 (-16.12, 18.27) | -0.17 (-18.90, 22.90) | -0.56 (-38.08, 59.70) | -0.91 (-27.06, 34.61) |
|  | Female | -0.35 (-21.82, 27.03) | -0.09 (-25.66, 34.27) | -0.25 (-48.69, 93.92) | -0.86 (-35.28, 51.86) |
|  | Male | -0.29 (-22.08, 27.59) | -0.02 (-25.72, 34.58) | -0.81 (-50.05, 97.00) | -0.94 (-36.57, 54.69) |
| New Zealand | Both | -4.89 (-5.65, -4.13) | -5.00 (-5.84, -4.16) | -5.06 (-8.11, -1.91) | -4.22 (-6.04, -2.36) |
|  | Female | -4.98 (-6.35, -3.59) | -5.28 (-6.86, -3.67) | -4.82 (-10.05, 0.71) | -4.02 (-6.78, -1.18) |
|  | Male | -5.04 (-5.99, -4.08) | -5.10 (-6.14, -4.05) | -5.36 (-9.15, -1.40) | -4.52 (-6.97, -1.99) |
| Niue | Both | -0.75 (-14.93, 15.80) | -0.45 (-17.12, 19.57) | -1.12 (-41.52, 67.20) | -1.47 (-26.10, 31.37) |
|  | Female | -0.94 (-20.15, 22.9) | -0.65 (-23.37, 28.80) | -1.19 (-52.78, 106.79) | -1.57 (-33.28, 45.22) |
|  | Male | -0.49 (-21.61, 26.33) | -0.17 (-24.86, 32.65) | -1.00 (-54.41, 115.00) | -1.31 (-36.87, 54.30) |
| Palau | Both | -0.65 (-8.60, 8.00) | -0.53 (-9.79, 9.69) | -0.46 (-20.74, 25.00) | -0.97 (-15.63, 16.25) |
|  | Female | -0.74 (-11.55, 11.40) | -0.66 (-13.02, 13.46) | -0.47 (-28.20, 37.96) | -0.98 (-21.53, 24.95) |
|  | Male | -0.53 (-12.29, 12.82) | -0.36 (-14.36, 15.92) | -0.42 (-28.27, 38.25) | -0.92 (-21.08, 24.38) |
| Papua New Guinea | Both | 0.72 (-0.14, 1.59) | 1.08 (-0.03, 2.02) | 0.53 (-2.20, 3.34) | 0.11 (-1.26, 1.50) |
|  | Female | 0.81 (-0.39, 2.02) | 1.14 (-0.39, 2.70) | 0.81 (-3.12, 4.91) | 0.27 (-1.66, 2.23) |
|  | Male | 0.63 (-0.62, 1.90) | 1.02 (-0.60, 2.67) | 0.28 (-3.50, 4.21) | -0.03 (-1.99, 1.96) |
| Philippines | Both | 1.60 (1.38, 1.83) | 1.76 (1.52, 1.99) | 1.52 (0.98, 2.05) | 1.30 (0.99, 1.62) |
|  | Female | 1.51 (1.24, 1.77) | 1.75 (1.42, 2.08) | 1.27 (0.52, 2.03) | 1.08 (0.65, 1.51) |
|  | Male | 1.70 (1.43, 1.97) | 1.79 (1.47, 2.12) | 1.75 (0.97, 2.53) | 1.51 (1.03, 2.00) |
| Republic of Korea | Both | -8.02 (-8.26, -7.78) | -7.14 (-7.48, -6.80) | -8.37 (-8.92, -7.81) | -8.81 (-9.15, -8.47) |
|  | Female | -9.18 (-9.49, -8.85) | -8.45 (-8.92, -7.98) | -9.52 (-10.35, -8.69) | -9.76 (-10.20, -9.32) |
|  | Male | -6.16 (-6.65, -5.67) | -5.40 (-6.08, -4.72) | -6.73 (-7.69, -5.76) | -6.93 (-7.64, -6.21) |
| Samoa | Both | -0.63 (-2.92, 1.71) | -0.36 (-3.14, 2.51) | -0.85 (-8.21, 7.10) | -1.22 (-5.19, 2.93) |
|  | Female | -0.35 (-3.37, 2.76) | -0.09 (-3.78, 3.74) | -0.43 (-10.40, 10.65) | -0.90 (-6.07, 4.57) |
|  | Male | -0.85 (-4.54, 2.98) | -0.53 (-5.05, 4.21) | -1.28 (-12.17, 10.96) | -1.55 (-7.88, 5.21) |
| Singapore | Both | -5.18 (-6.26, -4.08) | -4.84 (-6.10, -3.57) | -7.50 (-10.74, -4.14) | -6.31 (-8.47, -4.10) |
|  | Female | -5.96 (-7.48, -4.42) | -5.71 (-7.50, -3.88) | -7.65 (-12.18, -2.87) | -6.65 (-9.51, -3.69) |
|  | Male | -4.64 (-6.35, -2.89) | -4.31 (-6.28, -2.30) | -7.34 (-12.23, -2.18) | -5.95 (-9.49, -2.27) |
| Solomon Islands | Both | 0.02 (-2.85, 2.97) | 0.01 (-3.56, 3.71) | 0.34 (-7.69, 9.05) | 0.03 (-4.72, 5.01) |
|  | Female | 0.09 (-4.16, 4.53) | 0.09 (-5.48, 5.98) | 0.44 (-10.51, 12.71) | 0.09 (-6.36, 6.99) |
|  | Male | -0.06 (-3.90, 3.94) | -0.01 (-4.61, 4.81) | 0.04 (-11.35, 12.90) | -0.20 (-7.05, 7.16) |
| Tonga | Both | -0.35 (-4.37, 3.84) | -0.21 (-5.03, 4.86) | -0.12 (-12.87, 14.50) | -0.70 (-7.87, 7.03) |
|  | Female | -0.56 (-6.32, 5.57) | -0.40 (-7.70, 7.49) | -0.03 (-16.85, 20.20) | -0.81 (-9.92, 9.23) |
|  | Male | -0.21 (-6.05, 6.00) | -0.09 (-6.77, 7.06) | -0.26 (-19.27, 23.22) | -0.60 (-12.33, 12.70) |
| Tuvalu | Both | -0.76 (-10.20, 9.67) | -0.41 (-11.91, 12.58) | -0.68 (-26.92, 35.00) | -1.48 (-17.17, 17.19) |
|  | Female | -0.61 (-12.26, 12.58) | -0.30 (-14.47, 16.22) | -0.31 (-32.42, 47.04) | -1.23 (-20.33, 22.45) |
|  | Male | -0.91 (-17.09, 18.44) | -0.50 (-20.21, 24.09) | -1.15 (-41.04, 65.73) | -1.79 (-27.65, 33.32) |
| Vanuatu | Both | -0.28 (-4.15, 3.75) | -0.34 (-4.77, 4.92) | -0.50 (-11.04, 11.28) | -0.78 (-7.40, 6.31) |
|  | Female | -0.53 (-6.20, 5.49) | -0.31 (-7.21, 7.11) | -0.59 (-15.81, 17.38) | -0.94 (-10.58, 9.73) |
|  | Male | -0.06 (-5.29, 5.45) | 0.21 (-6.17, 7.02) | -0.46 (-14.50, 15.89) | -0.67 (-9.55, 9.09) |
| Viet Nam | Both | -0.70 (-0.83, -0.57) | -0.97 (-1.16, -0.77) | 0.43 (0.12, 0.74) | -0.50 (-0.67, -0.33) |
|  | Female | -1.07 (-1.24, -0.91) | -1.41 (-1.66, -1.16) | 0.21 (-0.23, 0.65) | -0.83 (-1.05, -0.61) |
|  | Male | -0.21 (-0.43, 0.02) | -0.38 (-0.70, -0.05) | 0.64 (0.15, 1.14) | -0.07 (-0.39, 0.25) |
| **Abbreviation:**CVD, cardiovascular disease. | | | | | |

**Supplementary Table 3.** The results of the Joinpoint regression analysis, showing the trends in age-standardized death rates (per 100,000 persons) for CVD attributable to secondhand smoke among both sexes, females and males in the world and six main regions from 1990 to 2019.

| Sex | Period | APC (95% CI) | *P* value | AAPC (95% CI) | *P* value | |
| --- | --- | --- | --- | --- | --- | --- |
| Global | | | | | | |
| Both | 1990–1994 | -0.67 (-1.06, -0.27) | 0.003 | -1.48 (-1.63, -1.33) | <0.001 | |
|  | 1994–1998 | -1.98 (-2.6, -1.35) | <0.001 |  |  |  |
|  | 1998–2003 | -0.93 (-1.34, -0.53) | <0.001 |  |  |  |
|  | 2003–2007 | -2.43 (-3.05, -1.8) | <0.001 |  |  |  |
|  | 2007–2014 | -1.89 (-2.11, -1.68) | <0.001 |  |  |  |
|  | 2014-2019 | -0.93 (-1.23, -0.63) | <0.001 |  |  |  |
| Female | 1990–1994 | -0.61 (-1.07, -0.14) | 0.015 | -1.58 (-1.75, -1.42) | <0.001 | |
|  | 1994–1998 | -2.11 (-2.81, -1.4) | <0.001 |  |  |  |
|  | 1998–2003 | -0.88 (-1.33, -0.43) | 0.001 |  |  |  |
|  | 2003–2008 | -2.72 (-3.16, -2.28) | <0.001 |  |  |  |
|  | 2008–2014 | -2.15 (-2.46, -1.84) | <0.001 |  |  |  |
|  | 2014–2019 | -0.8 (-1.13, -0.46) | <0.001 |  |  |  |
| Male | 1990–1994 | -0.75 (-1.11, -0.4) | 0.001 | -1.34 (-1.49, -1.19) | <0.001 | |
|  | 1994–1999 | -1.68 (-2.03, -1.32) | <0.001 |  |  |  |
|  | 1999–2003 | -0.9 (-1.46, -0.33) | 0.005 |  |  |  |
|  | 2003–2007 | -2.07 (-2.65, -1.49) | <0.001 |  |  |  |
|  | 2007–2017 | -1.45 (-1.56, -1.34) | <0.001 |  |  |  |
|  | 2017–2019 | -0.49 (-1.75, 0.78) | 0.414 |  |  |  |
| African Region | | | | | |  |
| Both | 1990–1999 | -0.05 (-0.16, 0.06) | 0.361 | -0.9 (-0.96, -0.84) | <0.001 | |
|  | 1999–2008 | -1.72 (-1.84, -1.59) | <0.001 |  |  |  |
|  | 2008–2019 | -0.93 (-1.01, -0.85) | <0.001 |  |  |  |
| Female | 1990–1999 | -0.28 (-0.42, -0.13) | 0.001 | -0.93 (-1.01, -0.85) | <0.001 | |
|  | 1999–2007 | -1.61 (-1.83, -1.4) | <0.001 |  |  |  |
|  | 2007–2019 | -0.95 (-1.05, -0.86) | <0.001 |  |  |  |
| Male | 1990–1994 | 0.06 (-0.22, 0.34) | 0.658 | -0.87 (-1, -0.74) | <0.001 | |
|  | 1994–1997 | 0.8 (-0.06, 1.67) | 0.066 |  |  |  |
|  | 1997–2000 | -1.03 (-1.92, -0.14) | 0.026 |  |  |  |
|  | 2000–2008 | -1.85 (-1.96, -1.73) | <0.001 |  |  |  |
|  | 2008–2019 | -0.91 (-0.97, -0.85) | <0.001 |  |  |  |
| Eastern Mediterranean Region | | | | | | |
| Both | 1990–1997 | 0.19 (0.05, 0.34) | 0.011 | -0.93 (-1, -0.86) | <0.001 | |
|  | 1997–2003 | -0.97 (-1.2, -0.74) | <0.001 |  |  |  |
|  | 2003–2012 | -1.77 (-1.88, -1.66) | <0.001 |  |  |  |
|  | 2012–2019 | -0.92 (-1.06, -0.77) | <0.001 |  |  |  |
| Female | 1990–1997 | 0.09 (-0.08, 0.26) | 0.292 | -1.03 (-1.12, -0.94) | <0.001 | |
|  | 1997–2003 | -1.17 (-1.44, -0.9) | <0.001 |  |  |  |
|  | 2003–2013 | -1.74 (-1.85, -1.63) | <0.001 |  |  |  |
|  | 2013–2019 | -0.99 (-1.21, -0.76) | <0.001 |  |  |  |
| Male | 1990–1995 | 0.65 (0.4, 0.9) | <0.001 | -0.79 (-0.86, -0.71) | <0.001 | |
|  | 1995–2003 | -0.6 (-0.75, -0.46) | <0.001 |  |  |  |
|  | 2003–2012 | -1.78 (-1.9, -1.66) | <0.001 |  |  |  |
|  | 2012–2019 | -0.73 (-0.89, -0.57) | <0.001 |  |  |  |
| European Region | | | | | | |
| Both | 1990–1994 | 1.8 (0.75, 2.85) | 0.002 | -2.17 (-2.49, -1.86) | <0.001 | |
|  | 1994–1998 | -2.86 (-4.42, -1.28) | 0.002 |  |  |  |
|  | 1998-2004 | -1.04 (-1.76, -0.32) | 0.008 |  |  |  |
|  | 2004–2014 | -4.51 (-4.79, -4.22) | <0.001 |  |  |  |
|  | 2014-2019 | -1.35 (-2.09, -0.61) | 0.001 |  |  |  |
| Female | 1990–1994 | 2.5 (1.32, 3.69) | <0.001 | -2.15 (-2.5, -1.79) | <0.001 | |
|  | 1994–1998 | -2.69 (-4.44, -0.92) | 0.006 |  |  |  |
|  | 1998-2004 | -1.05 (-1.85, -0.25) | 0.013 |  |  |  |
|  | 2004–2014 | -4.82 (-5.13, -4.5) | <0.001 |  |  |  |
|  | 2014-2019 | -1.22 (-2.06, -0.38) | 0.007 |  |  |  |
| Male | 1990–1994 | 0.91 (0, 1.83) | 0.05 | -2.24 (-2.52, -1.97) | <0.001 | |
|  | 1994–1998 | -3.09 (-4.47, -1.69) | <0.001 |  |  |  |
|  | 1998-2004 | -0.96 (-1.6, -0.32) | 0.006 |  |  |  |
|  | 2004–2013 | -4.29 (-4.59, -3.98) | <0.001 |  |  |  |
|  | 2013-2019 | -1.92 (-2.41, -1.43) | <0.001 |  |  |  |
| Region of the Americas | | | | | | |
| Both | 1990–1995 | -3.22 (-3.53, -2.92) | <0.001 | -3.2 (-3.38, -3.02) | <0.001 | |
|  | 1995–2002 | -4.34 (-4.57, -4.1) | <0.001 |  |  |  |
|  | 2002-2006 | -5.02 (-5.72, -4.31) | <0.001 |  |  |  |
|  | 2006-2010 | -3.67 (-4.4, -2.94) | <0.001 |  |  |  |
|  | 2010–2014 | -2.5 (-3.24, -1.76) | <0.001 |  |  |  |
|  | 2014-2019 | -0.27 (-0.61, 0.08) | 0.118 |  |  |  |
| Female | 1990–1996 | -2.89 (-3.14, -2.64) | <0.001 | -3.11 (-3.25, -2.97) | <0.001 | |
|  | 1996–2002 | -3.95 (-4.28, -3.62) | <0.001 |  |  |  |
|  | 2002-2007 | -5.09 (-5.56, -4.62) | <0.001 |  |  |  |
|  | 2007–2013 | -3.24 (-3.58, -2.89) | <0.001 |  |  |  |
|  | 2013-2019 | -0.65 (-0.92, -0.37) | <0.001 |  |  |  |
| Male | 1990–1995 | -3.48 (-3.79, -3.17) | <0.001 | -3.3 (-3.46, -3.15) | <0.001 | |
|  | 1995-2006 | -4.78 (-4.89, -4.67) | <0.001 |  |  |  |
|  | 2006-2010 | -3.66 (-4.38, -2.94) | <0.001 |  |  |  |
|  | 2010–2014 | -2.51 (-3.25, -1.76) | <0.001 |  |  |  |
|  | 2014-2019 | -0.14 (-0.49, 0.21) | 0.416 |  |  |  |
| South-East Asia Region | | | | | | |
| Both | 1990–2001 | -0.67 (-0.88, -0.45) | <0.001 | -0.87 (-1.19, -0.54) | <0.001 | |
|  | 2001–2004 | -2.77 (-5.78, 0.33) | 0.077 |  |  |  |
|  | 2004-2019 | -0.62 (-0.76, -0.49) | <0.001 |  |  |  |
| Female | 1990–2001 | -0.96 (-1.16, -0.76) | <0.001 | -1.08 (-1.56, -0.6) | <0.001 | |
|  | 2001-2004 | -3.53 (-6.24, -0.75) | 0.017 |  |  |  |
|  | 2004-2007 | -0.32 (-3.12, 2.56) | 0.816 |  |  |  |
|  | 2007–2010 | -2.29 (-5.09, 0.59) | 0.111 |  |  |  |
|  | 2010-2019 | -0.24 (-0.51, 0.04) | 0.084 |  |  |  |
| Male | 1990–2001 | -0.27 (-0.5, -0.04) | 0.023 | -0.58 (-0.98, -0.18) | 0.004 | |
|  | 2001-2004 | -2.69 (-5.83, 0.55) | 0.097 |  |  |  |
|  | 2004–2008 | 0.84 (-0.84, 2.56) | 0.311 |  |  |  |
|  | 2008-2019 | -0.83 (-1.07, -0.59) | <0.001 |  |  |  |
| Western Pacific Region | | | | | | |
| Both | 1990–1998 | -2.19 (-2.28, -2.09) | <0.001 | -1.43 (-1.56, -1.31) | <0.001 | |
|  | 1998–2004 | 0.39 (0.19, 0.59) | 0.001 |  |  |  |
|  | 2004-2007 | -3.05 (-3.89, -2.2) | <0.001 |  |  |  |
|  | 2007-2011 | -0.58 (-1.01, -0.15) | 0.012 |  |  |  |
|  | 2011-2015 | -2.47 (-2.89, -2.05) | <0.001 |  |  |  |
|  | 2015-2019 | -1.21 (-1.5, -0.92) | <0.001 |  |  |  |
| Female | 1990–1998 | -2.79 (-2.94, -2.64) | <0.001 | -1.89 (-2.07, -1.7) | <0.001 | |
|  | 1998-2004 | 0.2 (-0.1, 0.5) | 0.175 |  |  |  |
|  | 2004-2007 | -3.69 (-4.93, -2.43) | <0.001 |  |  |  |
|  | 2007–2011 | -1.52 (-2.15, -0.88) | <0.001 |  |  |  |
|  | 2011-2015 | -3.08 (-3.7, -2.46) | <0.001 |  |  |  |
|  | 2015-2019 | -0.96 (-1.43, -0.49) | 0.001 |  |  |  |
| Male | 1990–1998 | -1.13 (-1.23, -1.03) | <0.001 | -0.8 (-0.92, -0.68) | <0.001 | |
|  | 1998-2004 | 0.66 (0.46, 0.86) | <0.001 |  |  |  |
|  | 2004–2007 | -2.11 (-2.99, -1.23) | <0.001 |  |  |  |
|  | 2007-2011 | 0.56 (0.09, 1.03) | 0.023 |  |  |  |
|  | 2011-2019 | -1.72 (-1.83, -1.62) | <0.001 |  |  |  |
| **Abbreviation:** AAPC, average annual percent change; APC, annual percent change; CI, confidence interval. | | | | | | |

**Supplementary Figure 1.** The percentage of death number for CVD, ischemic heart disease, ischemic stroke and stroke in five groups (0-49, 50-54, 55-89, 90-94, >95 years) attributable to secondhand smoke for both sexes combined globally from 1990 to 2019.

**Abbreviation:** CVD, cardiovascular disease.

**Supplementary Figure 2.** The correlation of SDI levels with the age-standardized death rate for CVD caused by secondhand smoke in 27 countries from 1990 to 2019.

**Abbreviation:** SDI, socio-demographic index; CVD, cardiovascular disease.

**Supplementary Figure 3.** The temporal variation in the relative proportion of CVD deaths attributed to secondhand smoke across various age groups globally and in 27 countries and globally from 1990 to 2019.

**Abbreviation:** CVD, cardiovascular disease.

**Supplementary Figure 4.** The temporal variation in the relative proportion of ischemic heart disease deaths attributed to secondhand smoke across various age groups globally and in 27 countries from 1990 to 2019.

**Supplementary Figure 5.** The temporal variation in the relative proportion of ischemic stroke deaths attributed to secondhand smoke across various age groups globally and in 27 countries from 1990 to 2019.

**Supplementary Figure 6.** The temporal variation in the relative proportion of stroke deaths attributed to secondhand smoke across various age groups globally and in 27 countries from 1990 to 2019.

**Supplementary Figure 7.** The global temporal variation in the sex-specific relative proportion of CVD deaths attributed to secondhand smoke across various age groups from 1990 to 2019.

**Abbreviation:** CVD, cardiovascular disease.

**Supplementary Figure 8.** The temporal variation in the death rate for CVD caused by secondhand smoke across age groups globally and in 27 countries between 1990 and 2019.

The dumbbell plot's horizontal axis depicts "Deaths Rates, per 100,000."

**Abbreviation:** CVD, cardiovascular disease.

**Supplementary Figure 9.** The temporal variation in the death rate for ischemic heart disease caused by secondhand smoke across age groups globally and in 27 countries between 1990 and 2019.

The dumbbell plot's horizontal axis depicts "Deaths Rates, per 100,000."

**Supplementary Figure 10.** The temporal variation in the death rate for ischemic stroke caused by secondhand smoke across age groups globally and in 27 countries between 1990 and 2019.

The dumbbell plot's horizontal axis depicts "Deaths Rates, per 100,000."

**Supplementary Figure 11.** The temporal variation in the death rate for stroke caused by secondhand smoke across age groups globally and in 27 countries between 1990 and 2019.

The dumbbell plot's horizontal axis depicts "Deaths Rates, per 100,000."

**Supplementary Figure 12.** The temporal variation in the sex-specific deaths rate for CVD caused by secondhand smoke across age groups globally and in 27 countries between 1990 and 2019.

The dumbbell plot's horizontal axis depicts "Deaths Rates, per 100,000."

**Abbreviation:** CVD, cardiovascular disease.

**Supplementary Figure 13.** The local drifts of CVD deaths attributable to secondhand smoke globally and in 27 countries from 1990 to 2019.

**Abbreviation:** CVD, cardiovascular disease.

**Supplementary Figure 14.** The age effects of CVD deaths attributable to secondhand smoke globally and in 27 countries from 1990 to 2019.

**Abbreviation:** CVD, cardiovascular disease.

**Supplementary Figure 15.** The period effects of CVD deaths attributable to secondhand smoke globally and in 27 countries from 1990 to 2019.

**Abbreviation:** CVD, cardiovascular disease.

**Supplementary Figure 16.** The cohort effects of CVD deaths attributable to secondhand smoke globally and in 27 countries from 1990 to 2019.

**Abbreviation:** CVD, cardiovascular disease.

**Supplementary Figure 17.** The Joinpoint regression analysis of the age-standardized death rate for CVD attributable to secondhand smoke globally and in 27 countries from 1990 to 2019.

**Abbreviation:** AAPC, average annual percent change; APC, annual percent change; CVD, cardiovascular disease.
